# Understanding inequalities in access to adult mental health services in the UK: a systematic mapping review

**DOI:** 10.1186/s12913-023-10030-8

**Published:** 2023-09-29

**Authors:** Hayley J. Lowther-Payne, Anastasia Ushakova, Adelaide Beckwith, Catherine Liberty, Rhiannon Edge, Fiona Lobban

**Affiliations:** 1 Lancaster Medical School, Faculty of Health and Medicine, Health Innovation Campus, Lancaster University, Sir John Fisher Drive, Lancaster, Lancashire LA1 4AT UK; 2 Lancashire and South Cumbria NHS Foundation Trust, Sceptre Point, Sceptre Way, Bamber Bridge, Preston, Lancashire PR5 6AW UK; 3 Division of Health Research, Faculty of Health and Medicine, Health Innovation Campus, Lancaster University, Sir John Fisher Drive, Lancaster, LA1 4AT Lancashire UK

**Keywords:** Healthcare access, Inequalities, Mental health services, Systematic mapping review, United Kingdom

## Abstract

**Background:**

Population groups experience differential access to timely and high-quality mental healthcare. Despite efforts of recent UK policies to improve the accessibility of mental health services, there remains a lack of comprehensive understanding of inequalities in access to services needed to do this. This systematic mapping review aimed to address this gap by identifying which population groups continue to be poorly served by access to adult mental health services in the UK, how access has been measured, and what research methods have been applied.

**Methods:**

Seven electronic databases were searched from January 2014 up to May 2022. Primary research studies of any design were included if they examined access to adult NHS mental health services in the UK by population groups at risk of experiencing inequalities. Study characteristics, measures of access, inequalities studied, and key findings were extracted. A best-fit framework approach was used, applying Levesque’s Conceptual Framework for Healthcare Access to synthesise measures of access, and applying a template derived from Cochrane Progress-Plus and NHS Long Term Plan equality characteristics to synthesise key findings associated with inequalities.

**Results:**

Of 1,929 publications retrieved, 152 studies of various types were included. The most frequently considered dimensions of inequality were gender, age, and ethnicity, whilst social capital, religion, and sexual orientation were least frequently considered. Most studies researched access by measuring “healthcare utilisation”, followed by studies that measured “healthcare seeking”. Key barriers to access were associated with individuals’ “ability to seek” (e.g. stigma and discrimination) and “ability to reach” (e.g. availability of services). Almost half of the studies used routinely collected patient data, and only 16% of studies reported patient and public involvement.

**Conclusions:**

Little appears to have changed in the nature and extent of inequalities, suggesting that mental health services have not become more accessible. Actions to reduce inequalities should address barriers to population groups’ abilities to seek and reach services such as stigma-reducing interventions, and re-designing services and pathways. Significant benefits exist in using routinely collected patient data, but its limitations should not be ignored. More theoretically informed research, using a holistic measurement of access, is needed in this area.

**Review registration:**

10.17605/OSF.IO/RQ5U7.

**Supplementary Information:**

The online version contains supplementary material available at 10.1186/s12913-023-10030-8.

## Background

Mental ill health, such as depression, anxiety, and psychosis, is one of the top ten leading causes of global disease burden [1]. The World Health Organisation (WHO) 2022 report on “transforming mental health for all” called for action to strengthen global mental healthcare to address this need as services continue to be under-funded and under-resourced [2]. In 2016, it was estimated that only one in three people who experience a mental health condition in England could access the mental health support they need [3]. By 2021, an estimated 8 million people with mental health needs were not in contact with mental health services [4]. On the whole, individuals face high thresholds for being eligible to receive mental healthcare and if deemed eligible, long waiting times before receiving care [5]. Evidence suggests that population groups who have been exposed to social and economic disadvantage experience differential access to timely and high-quality mental healthcare in the UK [5].

Healthcare access however, is a complex concept to define and measure. Many theoretical frameworks have been developed to conceptualise access, adopting a range of ways to not only define what access is but also understand what may influence access. One of the most recent frameworks is Levesque’s Conceptual Framework for Healthcare Access [6], which views access as a multi-dimensional concept associated with dimensions of healthcare systems (e.g. their approachability), and individuals’ abilities to access healthcare (e.g. ability to seek). The application of theoretical frameworks is somewhat limited in mental health service research. The stigma people with mental health conditions experience and the existence of involuntary mental healthcare adds further complexity to understanding access to mental health services specifically. Given these unique challenges, there is a need to understand how existing research has conceptualised access in relation to mental healthcare.

In recent years, the UK Government have committed to improving the accessibility of publicly funded mental health services [7–9]. A recent report reviewing the progress of these commitments based on audits, suggests that whilst more people are now in contact with mental health services than in 2016, targets to improve access and address inequalities have been missed [4]. A comprehensive understanding of inequalities is required to review and improve access to mental health services for different population groups. The NHS Advancing Mental Health Equalities Strategy summarised differential access to mental health services across population group characteristics (e.g. age, ethnicity, deprivation, sexual orientation) [10]. Evidence drawn upon in this report however, was largely from the grey literature (e.g. third sector organisation reports). Reviewing the academic literature could develop a more empirical foundation to inform policy decision making and actions to address inequalities. Asthana et al. [11] conducted an evidence review, now 8 years old, of quantitative variations in access to NHS mental health services in England, and reported differences associated with age, gender, ethnicity, socioeconomic status, and geographical area. The review however, omitted other dimensions (e.g. sexual orientation, gender identity, refugee and asylum seeker status), did not review the intersectionality of these groups, and did not include qualitative evidence. Therefore, it is necessary to update these findings to not only consider more recent research (e.g. impact of COVID-19, effect of mental health policies), but also to consider other dimensions of inequalities and qualitative evidence that may be able to contextualise quantitative variations in access to mental health services between groups.

This systematic mapping review collated existing evidence to identify which population groups are poorly served by access to adult mental health services in the UK. The review explored how access was measured and which, if any, theoretical frameworks have been applied. Due to the complexity of mental health services across different countries and the unique challenges posed for insurance-based and universal healthcare systems, this review focused only on the UK context. The NHS Advancing Mental Health Strategy outlined the need to use data to drive insight and decision making to improve accessibility of services [10], so this review also assessed how routinely collected patient data has been used to quantify inequalities in access. Specifically, this systematic mapping review aimed to address the following research questions:

1. How has *access* been measured in research exploring inequalities in access to adult mental health services in the UK?
2. What *research methods* and *theoretical frameworks* have been applied in this research?
3. What evidence exists regarding the *differences in access* between population groups, and how does this evidence offer insights into inequalities in access to adult mental health services in the UK?
4. How has the analysis of *routinely collected patient data* from mental health services been used to understand inequalities in access?

## Methods

A systematic mapping review aims to map out and categorise existing evidence on a broader topic than would be studied in a typical systematic review, to develop an understanding of the literature and identify gaps that could be explored with further research [12]. Due to the breadth of evidence available in this area, the heterogeneity of studies, and the broad research questions, a systematic mapping review was deemed a suitable way of synthesising evidence from relevant studies. This review was conducted based on existing guidance for scoping reviews [13], and reported based on the Preferred Reporting Items for Systematic Reviews and Meta-Analyses extension for Scoping Reviews (PRISMA-ScR) checklist [14] (see Table S1 in Additional file 1 for reporting checklist), as one does not specifically exist for systematic mapping reviews.

### Search strategy

Studies were identified through searching the titles, abstracts, and keywords of records across seven electronic databases (Academic Search Ultimate via EBSCOhost, CINAHL via EBSCOhost, EMBASE via Ovid, MEDLINE Complete via EBSCOhost, PsycINFO via EBSCOhost, Scopus via Scopus, and Web of Science via Clarivate) from January 2014, in line with the release of the NHS Five Year Forward report [8] and to extend previous review findings [11], up to 25th May 2022. A search strategy using a combination of Subject Headings and keywords related to main concepts of the research questions was developed and finalised with the assistance of a Faculty Librarian from Lancaster University. Search terms used across all searches are presented in Table 1. Table S2 presents the search strategies used across the seven databases, the date the search was conducted, and the corresponding number of results identified (Additional file 2). Additional studies were identified through screening reference lists and citations of included studies and relevant review articles.

**Table 1:** Search terms

Key concepts	Search terms – combination used across all databases
Mental health services	mental health care OR mental healthcare OR mental health service* OR mental health therap* OR mental health treatment* OR psychological care OR psychological service* OR psychological therap* OR psychological treatment* OR psychiatric care OR psychiatric service* OR psychiatric therap* OR psychiatric treatment*
Access	access OR accessibility OR availability OR consultation* OR contact* OR entry OR pathway* OR referral* OR utilisation OR utilization OR use OR uptake
Inequalities	barrier* OR determinant* OR difference* OR disadvantage* OR discriminat* OR disparit* OR equal* OR equit* OR facilitator* OR inequal* OR inequit* or intersectional* OR minorit* OR unequal OR unfair OR variation*
UK	united kingdom OR uk OR great britain OR england OR wales OR scotland OR northern ireland OR national health service OR nhs OR london

### Eligibility criteria

Preliminary searches were used to develop the eligibility criteria. Primary research studies of any design (quantitative, qualitative, mixed methods) which examined access to adult mental health services in the UK and focused on population groups noted to be at risk of experiencing inequalities according to the NHS Long Term Plan [7] and Cochrane Progress-Plus framework [15] were eligible for inclusion. Studies were limited to those published in English. As grey literature (e.g. charity reports, policy documents) had already been summarised in a recent NHS policy document [10], these types of documents were not considered for inclusion. The eligibility criteria is outlined in Table 2.

**Table 2:** Eligibility criteria

Eligibility criteria	
**Include**	
Study type / design	Any primary research studies (quantitative, qualitative, mixed methods)
Setting / context	UK-based (England, Wales, Scotland, Northern Ireland)
Population(s) / participants	Adult populations (aged 18 +) noted to be at risk of experiencing inequalities according to NHS Long Term Plan [7] and Cochrane Progress-Plus framework [15]
Concept of access	Considers population groups that need to, have tried to, and/or have gained entry to adult mental health services in the UK
Mental health services	Specialist mental health service provision offered at primary, secondary, or tertiary levels of the National Health Service in the UK
Outcome measure(s)	Differences in or challenges to accessing adult mental health services between population groups (quantitative, qualitative)
Publication type	Peer-reviewed research articles
Publication date	From 1^st^ January 2014 to 25^th^ May 2022
Publication language	English
**Exclude**	
Population(s) / participants	Children and young people
Publication type	Review articles, letters, editorials, opinion pieces, study protocols, grey literature, conference abstracts

### Data selection

All retrieved citations from the searches were collated in EndNote [16] and duplicates were removed. The remaining citations were imported into Rayyan [17]. One reviewer (HL) screened titles and abstracts of retrieved citations against the eligibility criteria in Rayyan. Full texts of studies thought potentially relevant were obtained and assessed by HL. Twenty percent of the titles and abstracts, and 15% of full text articles were screened by a second reviewer (AB/CL) to check consistency and accuracy in applying eligibility criteria. Uncertainty or disagreements at any stage were resolved through discussion, and if consensus could not be reached, the wider review group was consulted. Reasons for exclusion at the full text screening stage were documented.

### Data charting and synthesis

A bespoke data extraction form was developed and piloted to collect relevant information from included studies. Data extracted included author(s), year of publication, study aim(s), setting, design, population, theoretical framework (if applicable), measure of access, measure of inequality, and key findings. Data extraction was performed by HL and a 5% sample of this was checked by a second reviewer (AB/CL) to verify completeness and accuracy. Any discrepancies were resolved through discussion or consultation with a third reviewer, and where necessary the wider review team. Quality assessment was not conducted in this review as studies were not going to be excluded on this basis.

Study characteristics (e.g. design, setting) were tabulated and synthesised narratively to describe the type of evidence available. A best-fit framework approach [18, 19] was used to analyse the data. Levesque’s Conceptual Framework for Healthcare Access [6] was used as the a priori framework to code how each study had measured access, applying the five stages of access as key concepts: perception of needs and desire for care, healthcare seeking, healthcare reaching, healthcare utilisation, and healthcare consequences. This framework offered a useful conceptualisation of access to healthcare as a multi-dimensional concept, and has not been used in this way in reviewing mental health service research.

A further framework was developed by combining equality characteristics in the NHS Long Term Plan [7], and the Cochrane Progress-Plus framework [15]: age, disability, education, gender and sex (including gender identity), occupation, place of residence, pregnancy/maternity, ethnicity, religion, sexual orientation, social capital, socioeconomic status, and other. This template was used as the a priori framework to identify which dimensions of inequality had been studied and to code key findings from the studies. Key findings for each dimension of the template framework were grouped together in the synthesis: differences in levels of access, differences in pathways to access, and barriers to accessing mental health services. For data related to barriers to access, the abilities of individuals to access healthcare according to Levesque’s framework [6], were used to code factors identified by studies that had influenced access: ability to perceive, ability to seek, ability to reach, ability to pay, ability to engage. Tables and figures have been used to characterise the evidence base identified. HL performed the data synthesis and the wider review team were consulted during the process to review and feedback on the presentation and interpretation of the results.

### Stakeholder involvement

The proposed research questions were reviewed by a service user group and a public adviser from a marginalised group with lived experience of accessing mental health services. Their involvement led to the inclusion of a theoretical framework [6] as a lens to further understand how studies have measured access. Three co-authors (AB/CL/FL) have experience and expertise in delivering mental health services to adults experiencing mental health conditions. Finally, the authors received feedback on the review findings and their interpretation from experts-by-experience and domain-experts.

## Results

**Fig. 1:**
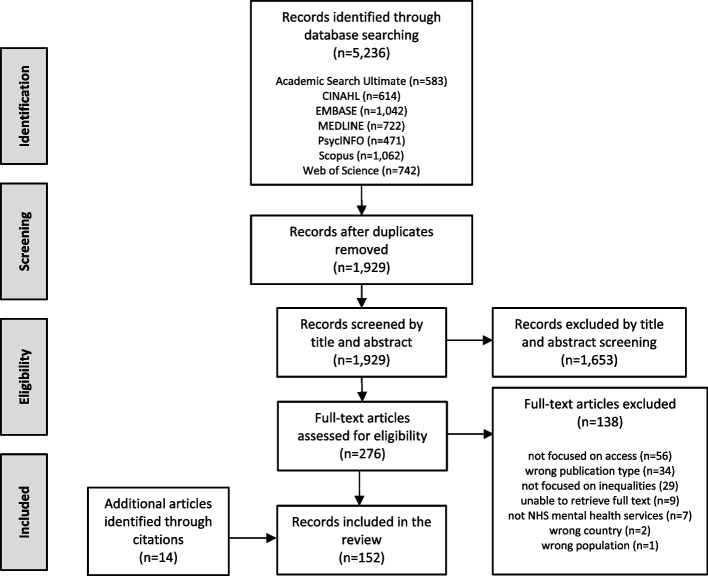
Flow chart of the study selection process

After the removal of duplicates, the search strategy identified a total of 1,929 citations. Based on screening titles and abstracts, 1,653 citations were excluded. A total of 276 full texts were assessed for eligibility, of which 138 papers were included in the review (Fig. 1). An additional 14 papers were also identified through citation checking.

### Study characteristics

An overview of the study characteristics is presented in Table 3, split by study type. The size of the literature on access to mental health services has grown gradually over time, seeing a larger increase in qualitative studies in more recent years. Over a third of studies were conducted in secondary care settings (e.g. community mental health teams, early intervention in psychosis services), and another third were conducted in other settings (e.g. population-based surveys, educational). The remaining studies were conducted across Improving Access to Psychological Therapies (IAPT) services, tertiary care (e.g. forensic services, veteran services), and primary care (e.g. GP) settings. Eighty percent of the studies were conducted in England, with fewer studies covering other nations in the UK (Wales (*n* = 6), Scotland (*n* = 4), Northern Ireland (*n* = 2), UK-wide (*n* = 24)). Of those conducted in England, nearly half of the studies were conducted in London (*n* = 50). Almost half of the studies used routinely collected patient data, 62 of which were quantitative. Only 25 studies reported any patient and public involvement, 15 of which were qualitative. Larger sample sizes were seen in quantitative studies.

**Table 3:** Summary of study characteristics

Study characteristic		Quantitative n (%)	Qualitative n (%)	Mixed n (%)	Total n (%)
Publication year	2014	9 (10)	3 (7)	1 (7)	13 (9)
	2015	9 (10)	2 (4)	3 (20)	14 (9)
	2016	8 (9)	2 (4)	2 (13)	12 (8)
	2017	12 (13)	6 (13)	1 (7)	19 (13)
	2018	8 (9)	3 (7)	1 (7)	12 (8)
	2019	14 (15)	2 (4)	2 (13)	18 (12)
	2020	13 (14)	6 (13)	1 (7)	20 (13)
	2021	12 (13)	12 (27)	1 (7)	25 (16)
	2022	7 (8)	9 (20)	3 (20)	19 (13)
Study setting	Primary care (e.g. GPs)	5 (5)	0 (0)	0 (0)	5 (3)
	IAPT services	10 (11)	4 (9)	5 (33)	19 (13)
	Secondary care (e.g. CMHTs)	42 (46)	12 (27)	1 (7)	55 (36)
	Tertiary (e.g. military, forensic)	5 (5)	2 (4)	2 (13)	9 (6)
	Other (e.g. educational)	27 (29)	25 (56)	7 (47)	59 (39)
	Multiple settings	3 (3)	2 (4)	0 (0)	5 (3)
Study design	Focus group	0 (0)	7 (16)	0 (0)	7 (5)
	Interview	8 (9)	34 (76)	1 (7)	43 (28)
	Observational	55 (60)	1 (2)	1 (7)	57 (38)
	Questionnaire / survey	20 (22)	2 (4)	10 (67)	32 (21)
	Multiple study designs	9 (10)	1 (2)	3 (20)	13 (9)
Study sample size	0 – 24	0 (0)	19 (42)	2 (13)	21 (14)
	25—150	7 (8)	26 (58)	6 (40)	39 (26)
	151—1,000	34 (37)	0 (0)	5 (33)	39 (26)
	1,001 – 10,000	27 (29)	0 (0)	0 (0)	27 (18)
	10,000 +	21 (23)	0 (0)	2 (13)	23 (15)
	Unclear / not stated	3 (3)	0 (0)	0 (0)	3 (2)
Evidence of PPI	Yes	4 (4)	15 (33)	6 (40)	25 (16)
	No	88 (96)	30 (67)	9 (60)	127 (84)
Use of routinely collected patient data	Yes	66 (72)	1 (2)	3 (20)	70 (46)
	No	26 (28)	44 (98)	12 (80)	82 (54)
Measuring access – using Levesque framework [6]	Perception of needs and desire for care	1 (1)	1 (2)	0 (0)	2 (1)
	Healthcare seeking	11 (12)	30 (67)	7 (47)	48 (32)
	Healthcare reaching	2 (2)	6 (13)	2 (13)	10 (7)
	Healthcare utilisation	77 (84)	7 (16)	6 (40)	90 (59)
	Healthcare consequences	1 (1)	1 (2)	0 (0)	2 (1)
Main dimensions of inequality studied	Age	12 (13)	5 (11)	3 (20)	20 (13)
	Disability	3 (3)	3 (7)	1 (7)	7 (5)
	Education	1 (1)	0 (0)	0 (0)	1 (1)
	Gender and sex	2 (2)	1 (2)	2 (13)	5 (3)
	Occupation	6 (7)	6 (13)	1 (7)	13 (9)
	Place of residence	1 (1)	0 (0)	0 (0)	1 (1)
	Pregnancy and maternity	2 (2)	1 (2)	1 (7)	4 (3)
	Race, ethnicity, culture, and language	19 (21)	17 (38)	1 (7)	37 (24)
	Religion	0 (0)	0 (0)	0 (0)	0 (0)
	Sexual orientation	1 (1)	1 (2)	1 (7)	3 (2)
	Social capital	0 (0)	0 (0)	0 (0)	0 (0)
	Socio-economic status	6 (7)	1 (2)	1 (7)	8 (5)
	^*^Contact with criminal justice system	7 (8)	1 (2)	0 (0)	8 (5)
	^*^Refugees and asylum seekers	1 (1)	2 (4)	0 (0)	3 (2)
	^*^Trafficked people and street sex workers	0 (0)	2 (4)	1 (7)	3 (2)
	Multiple / exploratory	31 (34)	5 (11)	3 (20)	39 (26)

*CMHTs* Community Mental Health Teams, *GP* General Practice, *IAPT* Improving Access to Psychological Therapies, *PPI* Patient and Public Involvement

### Measures of access

The five stages of access in Levesque’s framework [6] were used to note how each study measured access to mental health services. The superscript numbers used in this section refer to the references used in Additional file 3, which presents a table of included studies categorised by measure of access (Table S3).

#### Perception of needs and desire for care

Two studies^1−2^ explored illness perceptions and help-seeking attitudes of population groups and their influence on accessing mental health services. One study^1^ explored how illness attributions differed by ethnicity using a questionnaire, and another study^2^ interviewed service users about their perceptions of eligibility for mental healthcare during the COVID-19 pandemic.

#### Healthcare seeking

Healthcare seeking as a measure of access was used by 48 studies^3−50^. These were most notably qualitative studies^3−8,13,17,18,20–22,24,26–44,46,47,49,50^ which explored barriers to seeking mental healthcare from the perspectives of service users, carers, and professionals. Some quantitative studies which used routinely collected data^12,23^ or self-report surveys^9−11,14–16,19,25,45,48^ about being referred to mental health services were also included here as this suggested seeking mental healthcare but not necessarily reaching or utilising it. Most studies measuring healthcare seeking focused on a specific dimension of inequality, such as ethnicity^4−6,19,21,22,27,29,32,34–36,41,43,46,50^, and occupation^8,13,16,23,26,31,37,44,45,48,49^.

#### Healthcare reaching

Ten studies^51−60^ ascertained from service users or professionals, using mainly interviews, the barriers to reaching mental healthcare. Four studies^52−54,57^ were focused specifically on the dimension of disability and the availability and accommodation of mental health services (e.g. location, transport, mobility). Inadequate transitions from child and adolescent mental health services to adult mental health services were the focus of two studies^51,58^ measuring healthcare reaching.

#### Healthcare utilisation

Ninety studies^61−150^ measured healthcare utilisation, of which were mostly quantitative and observational. These studies either used routinely collected data or survey responses self-reporting use of mental health services to understand differences in rates of utilisation or receipt of care between population groups. Studies were predominantly conducted in secondary care or IAPT settings, most likely due to the routinely collected patient data that is available from these service providers. Twenty-eight^62,66,67,69,72–75,80,81,83,88,89,100,109,111,112,119,123,128,132,133,137,138,143,146,147^ studies measuring healthcare utilisation did not focus on a specific dimension of inequality and were mainly exploratory by looking at the characteristics of those accessing services, whilst 20 studies^61,63,65,68,77,85,88,94,99,103,104,113,114,120–122,124,135,136,150^ specifically focused on rates of utilisation by ethnicity.

#### Healthcare consequences

Two studies^151−152^ explored the consequences of accessing inappropriate mental healthcare. One study^151^ investigated the experiences of people with mental health conditions accessing remote mental healthcare during the COVID-19 pandemic, and another study^152^ examined unmet psychological care needs of people living with HIV and associated health outcomes.

### Research methods and theoretical frameworks

Quantitative studies (*n* = 92) were mostly observational using routinely collected patient data (*n* = 55), or surveys collecting quantitative data (*n* = 20), often using established scales (e.g. Barriers to Care, Stigma Scale), to examine differences between population groups. These studies had larger sample sizes and used sampling methods that were more representative, but were less likely to demonstrate evidence of patient and public involvement. Some quantitative studies combined minority groups due to small sample sizes (e.g. Black and minority ethnic, sexual minorities) assuming a shared experience. Descriptive statistics, statistical tests, such as Chi-square, and regression analyses were used to analyse differences between population groups. Qualitative studies (*n* = 45) were mainly interviews (*n* = 34) or focus groups (*n* = 7) conducted with service users, carers, or professionals about their experiences or perspectives on access to mental health services. Participants were recruited purposively, typically belonging to a particular minority group or professional role. Studies often used thematic analysis to synthesise the data, and were more likely to demonstrate evidence of patient and public involvement. Surveys collecting both quantitative and qualitative data were used in mixed methods studies (*n* = 10), but few studies referred to the integration of findings as would be seen in a typical mixed methods design. Only 17 studies discussed the application or production of a theoretical framework to understand access or inequality, and this was mostly frequently used to analyse qualitative data. Dixon-Woods’ Candidacy Framework [20], Andersen’s Model of Health Services Use [21], and Kleinman’s Healthcare Model [22], featured in multiple studies.

### Key findings on inequalities in access

**Fig. 2:**
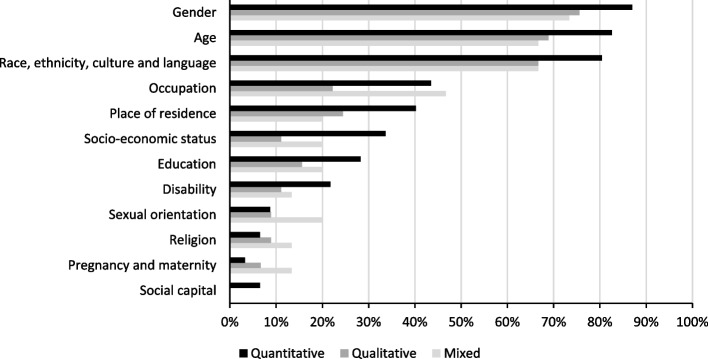
Percentage of studies that collected data for each dimension of inequality by study type

**Fig. 3:**
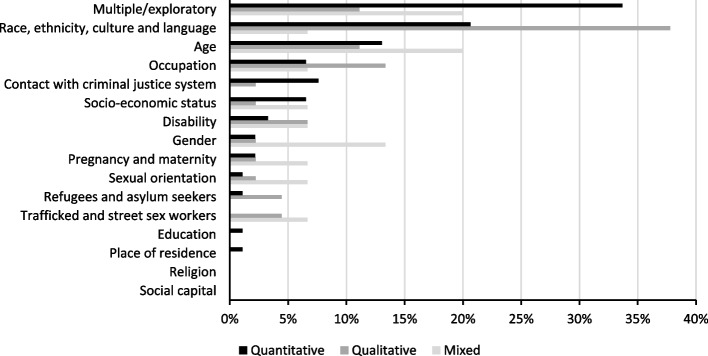
Main dimensions of inequality examined by the included studies by study type

To understand inequalities, data was most frequently collected by studies for gender (*n* = 125), age (*n* = 117), and ethnicity (*n* = 114). Social capital (*n* = 6), religion (*n* = 12), and sexual orientation (*n* = 15) were the least frequently considered. Figure 2 presents the percentage of studies that collected data for each dimension of inequality by study type. 113 studies focused on a specific dimension of inequality, these tended to use qualitative methods. Whilst the remaining studies (*n* = 39) were more exploratory or studied multiple dimensions of inequality, these tended to be quantitative. Figure 3 presents the percentage of studies that focused on a specific dimension of inequality by study type. Some studies only included specific groups in their study population, such as ethnic minorities (*n* = 17), young people (*n* = 11), and women in the pre-natal or post-natal period (*n* = 6).

The superscript numbers used in this section refer to the references used in Additional file 4, which presents a table of the key findings on inequalities in access by dimension of inequality (Table S4).

#### Differences in levels of access to mental health services

Forty-one studies found no differences in access between age groups^1−6^, disabilities^4,26,52,53^, educational qualifications^13,39^, gender and sex^1,3,4,6,13–15,20,23,24,26,30,32,33,35,38,66–68^, employment status^13,35,75^, place of residence^6,12,18,33,35^, ethnicity^1−3,6,11,14,30,33,38,53,76,92,97–100^, religion^3^, social capital^11,12^, socioeconomic status^16,18,26,35,75,76,128,135^, or relationship status^6,13,23,35^. Referral rates to secondary mental health services were found to be higher for young people^14^, people with long-term conditions^15^, females^16^, and lower for homeless people^53^, and those living in more deprived areas^136,137^. Access measured by mental health service contacts, admissions, and caseloads, highlighted a mixed picture of differences in access by age group, educational qualification, gender and sex, employment status, sexual orientation, and deprivation. Consistent findings for studies measuring access in this way were higher access for females^16,26,27,44,62,63,69,70^, unemployed people^29,44,49,62,76,77,78^, and prisoners^60,70,73^, and lower access for homeless people^53^, and ethnic minorities^13,24,26,27,44,62,64,77,102–107^. Working age adults^11^, people with long-term conditions^11^, those with higher educational qualifications^11−12^, females^10,11,61^, unemployed people^11^, those living alone^12^, people with a sense of belonging and social support^10^, those on lower incomes^11^, and single people^11^, were more likely to report formal mental health help-seeking (e.g. from a mental health professional). Higher mental health service costs were associated with younger and older adults^7−9^, people with long-term conditions^7,8^, males^8^, those living alone^7^, ethnic minorities^7^, and those living in more deprived areas^7−8^. Risk of disengagement with mental health treatment was found in younger adults^30^, people with learning disabilities^52^, unemployed people^30^, homeless people^53^, ethnic minority males^75^, Muslim males^75^, sexual minority males^75^, and males living in more deprived areas^75^. Unmet mental health needs were reported for people with disabilities^54^, people living with HIV^55^, males^70,72^, ethnic minorities^24,64^, and prisoners^64,72^.

#### Differences in pathways to access mental health services

Referral sources and destinations were explored by some studies to understand pathways into care. For IAPT services, GP-referred patients were more likely to be younger^29^, male^29^, unemployed^29^ and White^29^. There were little variation in IAPT access via self-referral routes. Black people^32,68,79,110–112^ and males^68^ had higher rates of criminal justice system involvement in their referral source to secondary mental health services. Despite presenting to primary care with psychological care needs, refugees and asylum seekers^145^, and migrants^62^ were unlikely to be referred to mental health services. Compulsory mental health treatment (e.g. being subject to a Mental Health Act section) was more likely for unemployed people^81^, those living alone^81^ or in supported accommodation^32^, ethnic minorities, particularly those from a Black ethnic background^34,79,81,105,110–113^, people from more deprived areas^34^, and single people^33^. Waiting times also differed amongst some groups with people from less deprived areas^6^, ethnic minorities^35^, and older people^28,31,35^ waiting less time for treatment.

#### Barriers to accessing mental health services

Barriers to accessing mental health services were most frequently associated with individuals’ “ability to reach” services, followed by individuals’ “ability to seek” services. Experiences of or anticipating experiences of stigma and discrimination was a key barrier to seeking mental health services across 43 studies, for age^39−42,44^, disability^55,56,58^, education^44,65^, gender and sex^61,65,69^, occupation^44,69,83–91^, pregnancy/maternity^95,96^, ethnicity^44,65,96,97,109,114–117,119–124,126–131^, sexual orientation^44,63,67,134^, contact with criminal justice system^97^, and refugee and asylum seeker status^146^. The majority of studies referred to stigma and discrimination related to having a mental health condition and/or accessing mental health services. However, for studies which looked specifically at ethnicity or sexual orientation, this barrier was also sometimes discussed in terms of individuals’ previous experiences of or anticipating future experiences of stigma and discrimination based on their identity as an ethnic minority^44,114–115,119,124,126–127,129^ or sexual minority^44,67,134^. Previous or anticipated experiences of racism or homophobia when accessing mental health services acted as barrier to seeking mental healthcare for these groups specifically. Thirty-two studies identified a key barrier to engaging with mental health services was the appropriateness of services to meet the needs of different population groups, for age^36,37,41^, disability^56,57^, gender and sex^71,74^, occupation^83,88,89^, place of residence^93^, pregnancy/maternity^94,95^, ethnicity^60,96,117,119,121,125,127,129,130,133^, sexual orientation^67,94,134^, socioeconomic status^141,143^, contact with criminal justice system^72,144^, trafficked people^147,149^, and street sex workers^148^. The availability of services was reported a barrier to reaching mental health services across 23 studies, for age^43,45,46^, disability^58,59^, occupation^83,85,87–89^, ethnicity^115,119,121,128,132,133^, socioeconomic status^141^, contact with criminal justice system^72,97,144^, refugees and asylum seekers^132^, trafficked people^147,149^, and street sex workers^148^. Difficulties in recognising mental health symptoms (*n* = 18) and trust in mental health professionals (*n* = 18) were barriers to perceiving mental health needs associated with age^39,49,43^, gender and sex^69,74^, occupation^69,83–90^, pregnancy/maternity^95^, ethnicity^114−120,122,124–128^, contact with criminal justice system^97^, and trafficked people^148^. No studies referred to barriers associated with individuals’ “ability to pay” for services, this is likely due to the provision of universal healthcare in the UK.

### Routinely collected patient data

Sixty-nine studies used routinely collected patient data, such as referrals, contacts, attendances, and admissions to mental health services, to explore differential rates of access between population groups. This frequently involved comparing access according to the patient demographic data available (e.g. age, gender, ethnicity, deprivation), and using descriptive statistics, statistical tests, and regression modelling to make inferences about how groups differ in rates of access. A few studies also analysed data such as referral source, referral destination, whether a contact was attended, and whether admission was voluntary, to understand pathways to care as a measure of access. Other data sources such as the UK Census or Office for National Statistics (ONS) data were used by some studies to examine whether access rates were proportionate with population estimates. However, the Census or ONS data tended to be out of date compared with the mental health service data. Other studies linked mental health service data with other health data, such as primary care data or community health survey data, to understand “potential access” (e.g. self-reporting a mental health need in a community health survey, GP appointment for mental health condition) and “realised access” (e.g. contact with a mental health service). A large proportion of studies that analysed routinely collected patient data, had used the Clinical Record Interactive Search (CRIS) system at South London and Maudsley NHS Foundation Trust (SLaM), a large mental health service provider, or had extracted data from NHS Digital, such as the IAPT service evaluation database. Almost all of the studies that used routinely collected patient data were coded as “healthcare utilisation”, as it was a direct quantification of individuals using mental health services. All studies discussed the usefulness of analysing routinely collected patient data to understand differences in access to mental health services, but also reflected on the challenges it poses when being used for research purposes. Its accuracy and completeness, particularly in relation to demographic data such as ethnicity and sexual orientation, incompleteness of which can limit understanding of inequalities, was the main challenge noted by study authors (*n* = 22).

## Discussion

This systematic mapping review synthesised research on inequalities in access to adult mental health services in the UK, and the measures of access, research methods, and key findings of relevant studies. It was important to update previous review findings [11], following the COVID-19 pandemic [23] and recent changes to UK policies [7–9]. Although there was significant heterogeneity amongst studies, this review has provided a broad overview of the evidence base through categorising studies by their approach to measuring access, and the dimensions of inequality that have been studied.

### Measures of access and research methods

Whilst this review found studies across the continuum of access as defined by Levesque’s framework [6], most were positioned in exploring healthcare utilisation. This is similar to findings from reviewing studies of other types of healthcare access [24]. Healthcare utilisation is determined by the need for care and whether healthcare can be accessed. However, this review found that accounting for differences in need was not routinely considered, and represents a deficiency in current ability to accurately understand inequalities in access to mental health services. This is a conclusion that was shared by Asthana et al. [11]. Levesque et al. [6] suggested that to understand the complexity of access, mixed methods research in different contexts is needed to ameliorate factors that influence access and develop strategies to improve access. This review has highlighted that there continues to be a paucity of theoretically informed evidence in this area, and studies tend to rely on a simple conceptualisation of access. Despite the valuable perspective that patients, carers, and the public can bring to research [25], their involvement was largely absent from this evidence base. There is a need to address challenges associated with involving patients, carers, and the public, and identify ways in which this can be reported effectively in the future [26].

### Inequalities in access to adult mental health services in the UK

This review reiterates findings from the previous review [11], suggesting that the evidence base of variations in access to mental health services remains complex and somewhat contradictory. Despite the implementation of policy changes, this review has highlighted that inequalities in access may persist for some population groups, such as ethnic minorities and older people. Studies published since 2014 did not indicate a consistent pattern of differences in access, finding over-representation of groups in some contexts (e.g. ethnic minorities and males in compulsory mental health treatment) and less access in others (e.g. ethnic minorities and males in IAPT services). These mixed findings could reflect the differences in which these services are accessed and the stages at which they are accessed. For example, a lack of access to lower intensity therapies such as those delivered by IAPT services could be associated with later presentation to compulsory mental health treatment if mental health conditions have deteriorated. These mixed findings could also highlight the importance of intersectionality in the context of inequalities [27]. For example, Smyth et al. [28] explored males accessing IAPT services, and reported differential access within the study population across other dimensions, such as ethnicity and sexual orientation. Differences in access may be obscured if studies do not consider variation within population groups. Despite considering additional dimensions of inequality beyond the scope of Asthana et al. [11], this review found that studies continued to focus on differences based on age, gender, and ethnicity. This is likely due to the data available from healthcare services for these characteristics. The absence of evidence of inequalities across dimensions such as religion, sexual orientation, and social capital, does not indicate that inequalities do not exist; and highlights a poor understanding of the extent of inequalities in access to mental health services in the UK for these population groups.

Unlike the previous review [11], qualitative data was analysed to identify key barriers to accessing mental health services across dimensions of inequalities. These findings have added some context to the factors that may influence access to mental health services for different population groups. Stigma and discrimination, appropriateness of services, availability of services, difficulties associated with recognising mental health problems, and trust, were frequently cited by studies; all of which are reflected in the wider literature on barriers to healthcare access [29–31]. The Health Stigma and Discrimination framework [32] theorises the mechanisms through which mental health-related stigma and discrimination influence access to healthcare services and how individuals with intersecting stigma, such as minority groups, can lead to a double burden. Action to reduce inequalities should consider how to address the barriers identified. Stigma-reducing interventions may be effective for specific population groups (e.g. ethnic minorities, LGBTQ + groups), such as individual support to overcome internalised stigma, or community support to change harmful attitudes towards mental ill health [32]. Re-designing services and pathways, in collaboration with population groups experiencing inequalities [25], could improve the accessibility and appropriateness of mental healthcare to meet the needs of different groups. Mental health awareness campaigns and community outreach programmes, particularly targeted at groups who have difficulties in recognising mental health need and trusting mental health professionals (e.g. veterans, ethnic minorities, LGBTQ + groups), could remove barriers to seeking mental healthcare [31].

### Routinely collected patient data

There are significant benefits to using routinely collected patient data to understand inequalities in access to mental health services. Primarily the data, particularly from secondary care services, has been used to examine differences in mental healthcare utilisation between population groups. Other studies had used data to identify variations in pathways into mental healthcare, or risk of disengaging from mental health treatment. Increases in the availability and accessibility of healthcare data have dramatically changed the landscape of population health research [33], presenting opportunities to conduct studies which require much less resource than primary data collection, and have real-world generalisability, often with large sample sizes [34]. There are challenges to overcome in using this data for research purposes, many of which study authors alluded to. Low quality or missingness of data on patient characteristics can influence our understanding of variations in access for population groups and limits what conclusions can be reached. As such, there may be hidden inequalities as a result of poor data collection and quality. Recent NHS Digital guidance [35] has set out to improve data quality for many of the dimensions of inequalities identified in this review, through enabling patient self-reporting, embedding inclusive ways of working and reducing staff assumptions, and sharing feedback on data quality. These planned improvements will enhance the use of this data to generate more reliable evidence of inequalities in access to mental health services and may clarify inconsistent findings.

### Strengths and limitations of the review

This systematic mapping review was conducted in line with existing guidelines for reviews [13], applied a well-established framework in the analysis [6], and included stakeholder involvement. Comprehensive searches were undertaken across seven electronic databases and eligibility criteria was kept intentionally broad to ensure relevant studies were included. Grey literature was not considered for inclusion in this review as it has been summarised elsewhere [10]. Whilst this review aimed to identify studies primarily focused on examining access, evidence from studies where this was not the primary focus and inadvertently found inequalities in access may have been missed. As this review captured a breadth of evidence rather than a specific standard of evidence, issues associated with quality appraisal were not addressed. This may have led to an oversimplification of concepts and could limit conclusions about the reliability of findings. There may also have been a publication bias in that studies where no differences or inequalities were found may be less likely to have been published than those that did. This review was unable to draw on the influence of mental health conditions and sometimes the service due to poor description available in the studies; this is important to assess in future studies as access and inequalities are likely to differ based on the condition experienced and the service accessed. This review was limited to studies conducted with adult populations accessing mental health services in the UK; additional insight of other contexts and for children and young people may be beneficial. The majority of the studies identified were conducted in England, particularly London, and so there is a potential limitation to the review findings being generalisable to other regions in England and in the UK. Further exploration to understand inequalities in access to mental health services within these contexts is needed.

## Conclusion

This systematic mapping review successfully applied an established framework to synthesise a large heterogenous body of research on inequalities in access to adult mental health services in the UK. The findings indicate that attempts to understand inequalities in access to mental health services require a much more holistic measurement of access than being used in current research. Little has changed in the nature and extent of inequalities, suggesting mental health services have not become more accessible as was planned in policy. Whilst using routinely collected data to measure mental healthcare utilisation provides a useful contribution to understanding inequalities, relying solely on quantifying if someone uses a mental health service does not present an opportunity to fully understand the complexities of access. Policy on addressing inequalities in access to mental health services could be better informed by mixed methods research which attempts to contextualise access in a holistic way, such as considering mental health need, help-seeking behaviour, and healthcare utilisation.

### Supplementary Information

**Additional file 1: Table S1. **Preferred Reporting Items for Systematic reviews and Meta-Analyses extension for Scoping Reviews (PRISMA-ScR) Checklist.

**Additional file 2: Table S2. **Search strategies.

**Additional file 3: Table S3. **Summary of included studies.

**Additional file 4: Table S4. **Summary of key findings associated with dimensions of inequality

## Data Availability

All data generated or analysed as part of this review are included in this publication and its supplementary information files.
