# Supplementary material for: Understanding inequalities in access to adult mental health services in the UK: a systematic mapping review
Source: BMC Health Serv Res. 2023 Sep 29;23:1042. doi: 10.1186/s12913-023-10030-8 (PMC10542667; doi:10.1186/s12913-023-10030-8)
Supplement: Supplementary file 2 — Additional file 2: Table S2. Search strategies. [file 12913_2023_10030_MOESM2_ESM.docx]

**Additional file 2**

**Table S2.** Search strategies

| **Database (platform)** | **Search number** | **Search string** | **Number of results (date search conducted)** |
| --- | --- | --- | --- |
| Academic Search Ultimate (EBSCOhost) | S1 | ( DE “MENTAL health services” ) OR ( TI ( “mental health care” OR “mental healthcare” OR “mental health service*” OR “mental health therap*” OR “mental health treatment*” OR “psychological care” OR “psychological service*” OR “psychological therap*” OR “psychological treatment*” OR “psychiatric care” OR “psychiatric service*” OR “psychiatric therap*” OR “psychiatric treatment*” ) ) OR ( AB ( “mental health care” OR “mental healthcare” OR “mental health service*” OR “mental health therap*” OR “mental health treatment*” OR “psychological care” OR “psychological service*” OR “psychological therap*” OR “psychological treatment*” OR “psychiatric care” OR “psychiatric service*” OR “psychiatric therap*” OR “psychiatric treatment*” ) ) | 583 results (25/05/2022) |
|  | S2 | ( ( DE “HEALTH services accessibility” ) OR ( DE “MENTAL health services use” ) ) OR ( TI ( “access” OR “accessibility” OR “availability” OR “consultation*” OR “contact*” OR “entry” OR “pathway*” OR “referral*” OR “utilisation” OR “utilization” OR “use” OR “uptake” ) ) OR ( AB ( “access” OR “accessibility” OR “availability” OR “consultation*” OR “contact*” OR “entry” OR “pathway*” OR “referral*” OR “utilisation” OR “utilization” OR “use” OR “uptake” ) ) |  |
|  | S3 | ( ( DE “HEALTH equity” ) OR ( DE “DISCRIMINATION in medical care” ) OR ( DE “MEDICAL care of minorities” ) ) OR ( TI ( “barrier*” OR “determinant*” OR “difference*” OR “disadvantage*” OR “discriminat*” OR “disparit*” OR “equal*” OR “equit*” OR “facilitator*” OR “inequal*” OR “inequit*” OR “intersectional*” OR “minorit*” OR “unequal” OR “unfair” OR “variation*” ) ) OR ( AB ( “barrier*” OR “determinant*” OR “difference*” OR “disadvantage*” OR “discriminat*” OR “disparit*” OR “equal*” OR “equit*” OR “facilitator*” OR “inequal*” OR “inequit*” OR “intersectional*” OR “minorit*” OR “unequal” OR “unfair” OR “variation*” ) ) |  |
|  | S4 | ( TI ( “united kingdom” OR “uk” OR “england” OR “wales” OR “scotland” OR “northern ireland” OR “national health service” OR “nhs” OR “london” ) ) OR ( AB ( “united kingdom” OR “uk” OR “england” OR “wales” OR “scotland” OR “northern ireland” OR “national health service” OR “nhs” OR “london” ) ) |  |
|  | S1 AND S2 AND S3 AND S4 | Limited to English language  Limited to 2014+ |  |
| CINAHL (EBSCOhost) | S1 | ( ( MH “Mental Health Services+” ) OR ( MH “Community Mental Health Services” ) ) OR ( TI ( “mental health care” OR “mental healthcare” OR “mental health service*” OR “mental health therap*” OR “mental health treatment*” OR “psychological care” OR “psychological service*” OR “psychological therap*” OR “psychological treatment*” OR “psychiatric care” OR “psychiatric service*” OR “psychiatric therap*” OR “psychiatric treatment*” ) ) OR ( AB ( “mental health care” OR “mental healthcare” OR “mental health service*” OR “mental health therap*” OR “mental health treatment*” OR “psychological care” OR “psychological service*” OR “psychological therap*” OR “psychological treatment*” OR “psychiatric care” OR “psychiatric service*” OR “psychiatric therap*” OR “psychiatric treatment*” ) ) | 614 results (25/05/2022) |
|  | S2 | ( ( MH “Health Services Accessibility+” ) OR ( MH “Referral and Consultation+” ) ) OR ( TI ( “access” OR “accessibility” OR “availability” OR “consultation*” OR “contact*” OR “entry” OR “pathway*” OR “referral*” OR “utilisation” OR “utilization” OR “use” OR “uptake” ) ) OR ( AB ( “access” OR “accessibility” OR “availability” OR “consultation*” OR “contact*” OR “entry” OR “pathway*” OR “referral*” OR “utilisation” OR “utilization” OR “use” OR “uptake” ) ) |  |
|  | S3 | ( ( MH “Healthcare Disparities” ) OR ( MH “Social Determinants of Health” ) ) OR ( TI ( “barrier*” OR “determinant*” OR “difference*” OR “disadvantage*” OR “discriminat*” OR “disparit*” OR “equal*” OR “equit*” OR “facilitator*” OR “inequal*” OR “inequit*” OR “intersectional*” OR “minorit*” OR “unequal” OR “unfair” OR “variation*” ) ) OR ( AB ( “barrier*” OR “determinant*” OR “difference*” OR “disadvantage*” OR “discriminat*” OR “disparit*” OR “equal*” OR “equit*” OR “facilitator*” OR “inequal*” OR “inequit*” OR “intersectional*” OR “minorit*” OR “unequal” OR “unfair” OR “variation*” ) ) |  |
|  | S4 | ( MH “United Kingdom” ) OR ( TI ( “united kingdom” OR “uk” OR “england” OR “wales” OR “scotland” OR “northern ireland” OR “national health service” OR “nhs” OR “london” ) ) OR ( AB ( “united kingdom” OR “uk” OR “england” OR “wales” OR “scotland” OR “northern ireland” OR “national health service” OR “nhs” OR “london” ) ) |  |
|  | S1 AND S2 AND S3 AND S4 | Limited to English language  Limited to 2014+ |  |
| EMBASE (Ovid) | S1 | 1. “mental health care”.ab,kw,ti. 2. “mental healthcare”.ab,kw,ti. 3. “mental health service*””.ab,kw,ti. 4. “mental health therap*”.ab,kw,ti. 5. “mental health treatment*”.ab,kw,ti. 6. “psychological care”.ab,kw,ti. 7. “psychological service*”.ab,kw,ti. 8. “psychological therap*”.ab,kw,ti. 9. “psychological treatment*”.ab,kw,ti. 10. “psychiatric care”.ab,kw,ti. 11. “psychiatric service*”.ab,kw,ti. 12. “psychiatric therap*”.ab,kw,ti. 13. “psychiatric treatment*”.ab,kw,ti. 14. exp mental health service/ 15. exp community mental health service 16. 1 or 2 or 3 or 4 or 5 or 6 or 7 or 8 or 9 or 10 or 11 or 12 or 13 or 14 or 15 | 1,042 results (25/05/2022) |
|  | S2 | 1. “access”.ab,kw,ti. 2. “accessibility”.ab,kw,ti. 3. “availability”.ab,kw,ti. 4. “consultation*”.ab,kw,ti. 5. “contact*”.ab,kw,ti. 6. “entry”.ab,kw,ti. 7. “pathway*”.ab,kw,ti. 8. “referral*”.ab,kw,ti. 9. “utilisation”.ab,kw,ti. 10. “utilization”.ab,kw,ti. 11. “use”.ab,kw,ti. 12. “uptake”.ab,kw,ti. 13. exp health care access/ 14. exp patient referral/ 15. 17 or 18 or 19 or 20 or 21 or 22 or 23 or 24 or 25 or 26 or 27 or 28 or 29 or 30 |  |
|  | S3 | 1. “barrier*”.ab,kw,ti. 2. “determinant*”.ab,kw,ti. 3. “difference*”.ab,kw,ti. 4. “disadvantage*”.ab,kw,ti. 5. “discriminat*”.ab,kw,ti. 6. “disparit*”.ab,kw,ti. 7. “equal*”.ab,kw,ti. 8. “equit*”.ab,kw,ti. 9. “facilitator*”.ab,kw,ti. 10. “inequal*”.ab,kw,ti. 11. “inequit*”.ab,kw,ti. 12. “intersectional*”.ab,kw,ti. 13. “minorit*”.ab,kw,ti. 14. “unequal” .ab,kw,ti. 15. “unfair” .ab,kw,ti. 16. “variation*”.ab,kw,ti. 17. exp health care disparity/ 18. exp health disparity/ 19. exp social inequality/ 20. exp social determinants of health/ 21. 32 or 33 or 34 or 35 or 36 or 37 or 38 or 39 or 40 or 41 or 42 or 43 or 44 or 45 or 46 or 47 or 48 or 49 or 50 or 51 |  |
|  | S4 | 1. “united kingdom”.ab,kw,ti. 2. “uk”.ab,kw,ti. 3. “england”.ab,kw,ti. 4. “wales”.ab,kw,ti. 5. “scotland”.ab,kw,ti. 6. “northern ireland”.ab,kw,ti. 7. “national health service”.ab,kw,ti. 8. “nhs”.ab,kw,ti. 9. “london”.ab.kw.ti 10. exp united kingdom/ 11. 53 or 54 or 55 or 56 or 57 or 58 or 59 or 60 or 61 or 62 |  |
|  | S1 AND S2 AND S3 AND S4 | 1. 16 and 31 and 52 and 63 2. limit to (english language and yr=”2014 -Current”) |  |
| MEDLINE Complete (EBSCOhost) | S1 | ( ( MH “Mental Health Services+” ) OR ( MH “Community Mental Health Services” ) ) OR ( TI ( “mental health care” OR “mental healthcare” OR “mental health service*” OR “mental health therap*” OR “mental health treatment*” OR “psychological care” OR “psychological service*” OR “psychological therap*” OR “psychological treatment*” OR “psychiatric care” OR “psychiatric service*” OR “psychiatric therap*” OR “psychiatric treatment*” ) ) OR ( AB ( “mental health care” OR “mental healthcare” OR “mental health service*” OR “mental health therap*” OR “mental health treatment*” OR “psychological care” OR “psychological service*” OR “psychological therap*” OR “psychological treatment*” OR “psychiatric care” OR “psychiatric service*” OR “psychiatric therap*” OR “psychiatric treatment*” ) ) | 722 results (25/05/2022) |
|  | S2 | ( ( MH “Health Services Accessibility+” ) OR ( MH “Referral and Consultation+” ) ) OR ( TI ( “access” OR “accessibility” OR “availability” OR “consultation*” OR “contact*” OR “entry” OR “pathway*” OR “referral*” OR “utilisation” OR “utilization” OR “use” OR “uptake” ) ) OR ( AB ( “access” OR “accessibility” OR “availability” OR “consultation*” OR “contact*” OR “entry” OR “pathway*” OR “referral*” OR “utilisation” OR “utilization” OR “use” OR “uptake” ) ) |  |
|  | S3 | ( ( MH “Healthcare Disparities” ) OR ( MH “Health Inequities+” ) OR ( MH “Social Determinants of Health” ) ) OR ( TI ( “barrier*” OR “determinant*” OR “difference*” OR “disadvantage*” OR “discriminat*” OR “disparit*” OR “equal*” OR “equit*” OR “facilitator*” OR “inequal*” OR “inequit*” OR “intersectional*” OR “minorit*” OR “unequal” OR “unfair” OR “variation*” ) ) OR ( AB ( “barrier*” OR “determinant*” OR “difference*” OR “disadvantage*” OR “discriminat*” OR “disparit*” OR “equal*” OR “equit*” OR “facilitator*” OR “inequal*” OR “inequit*” OR “intersectional*” OR “minorit*” OR “unequal” OR “unfair” OR “variation*” ) ) |  |
|  | S4 | ( MH “United Kingdom” ) OR ( TI ( “united kingdom” OR “uk” OR “great britain” OR “england” OR “wales” OR “scotland” OR “northern ireland” OR “national health service” OR “nhs” OR “london” ) ) OR ( AB ( “united kingdom” OR “uk” OR “england” OR “wales” OR “scotland” OR “northern ireland” OR “national health service” OR “nhs” OR “london” ) ) |  |
|  | S1 AND S2 AND S3 AND S4 | Limited to English language  Limited to 2014+ |  |
| PsycINFO (EBSCOhost) | S1 | ( ( DE “Mental Health Services” ) OR ( DE “Community Mental Health Services” ) ) OR ( TI ( “mental health care” OR “mental healthcare” OR “mental health service*” OR “mental health therap*” OR “mental health treatment*” OR “psychological care” OR “psychological service*” OR “psychological therap*” OR “psychological treatment*” OR “psychiatric care” OR “psychiatric service*” OR “psychiatric therap*” OR “psychiatric treatment*” ) ) OR ( AB ( “mental health care” OR “mental healthcare” OR “mental health service*” OR “mental health therap*” OR “mental health treatment*” OR “psychological care” OR “psychological service*” OR “psychological therap*” OR “psychological treatment*” OR “psychiatric care” OR “psychiatric service*” OR “psychiatric therap*” OR “psychiatric treatment*” ) ) | 471 results (25/05/2022) |
|  | S2 | ( ( DE “Health Care Access” ) OR ( DE “Health Care Utilization” ) ) OR ( TI ( “access” OR “accessibility” OR “availability” OR “consultation*” OR “contact*” OR “entry” OR “pathway*” OR “referral*” OR “utilisation” OR “utilization” OR “use” OR “uptake” ) ) OR ( AB ( “access” OR “accessibility” OR “availability” OR “consultation*” OR “contact*” OR “entry” OR “pathway*” OR “referral*” OR “utilisation” OR “utilization” OR “use” OR “uptake” ) ) |  |
|  | S3 | ( ( DE “Health Disparities” ) OR ( DE “Mental Health Disparities” ) OR ( DE “Mental Health Stigma” ) ) OR ( TI ( “barrier*” OR “determinant*” OR “difference*” OR “disadvantage*” OR “discriminat*” OR “disparit*” OR “equal*” OR “equit*” OR “facilitator*” OR “inequal*” OR “inequit*” OR “intersectional*” OR “minorit*” OR “unequal” OR “unfair” OR “variation*” ) ) OR ( AB ( “barrier*” OR “determinant*” OR “difference*” OR “disadvantage*” OR “discriminat*” OR “disparit*” OR “equal*” OR “equit*” OR “facilitator*” OR “inequal*” OR “inequit*” OR “intersectional*” OR “minorit*” OR “unequal” OR “unfair” OR “variation*” ) ) |  |
|  | S4 | ( TI ( “united kingdom” OR “uk” OR “england” OR “wales” OR “scotland” OR “northern ireland” OR “national health service” OR “nhs” OR “london” ) ) OR ( AB ( “united kingdom” OR “uk” OR “england” OR “wales” OR “scotland” OR “northern ireland” OR “national health service” OR “nhs” OR “london” ) ) |  |
|  | S1 AND S2 AND S3 AND S4 | Limited to English language  Limited to 2014+ |  |
| Scopus (Scopus) | S1 | TITLE-ABS-KEY ( “mental health care” OR “mental healthcare” OR “mental health service*” OR “mental health therap*” OR “mental health treatment*” OR “psychological care” OR “psychological service*” OR “psychological therap*” OR “psychological treatment*” OR “psychiatric care” OR “psychiatric service*” OR “psychiatric therap*” OR “psychiatric treatment*” ) | 1,062 results (27/05/2022) |
|  | S2 | TITLE-ABS-KEY ( “access” OR “accessibility” OR “availability” OR “consultation*” OR “contact*” OR “entry” OR “pathway*” OR “referral*” OR “utilisation” OR “utilization” OR “use” OR “uptake” ) |  |
|  | S3 | TITLE-ABS-KEY ( “barrier*” OR “determinant*” OR “difference*” OR “disadvantage*” OR “discriminat*” OR “disparit*” OR “equal*” OR “equit*” OR “facilitator*” OR “inequal*” OR “inequit*” OR “intersectional*” OR “minorit*” OR “unequal” OR “unfair” OR “variation*” ) |  |
|  | S4 | TITLE-ABS-KEY ( “united kingdom” OR “uk” OR “england” OR “wales” OR “scotland” OR “northern ireland” OR “national health service” OR “nhs” OR “london” ) |  |
|  | S1 AND S2 AND S3 AND S4 | Limited to English language  Limited to 2014+ |  |
| Web of Science (Clarivate) | S1 | TS= ( “mental health care” OR “mental healthcare” OR “mental health service*” OR “mental health therap*” OR “mental health treatment*” OR “psychological care” OR “psychological service*” OR “psychological therap*” OR “psychological treatment*” OR “psychiatric care” OR “psychiatric service*” OR “psychiatric therap*” OR “psychiatric treatment*” ) | 742 results (25/05/2022) |
|  | S2 | TS= ( “access” OR “accessibility” OR “availability” OR “consultation*” OR “contact*” OR “entry” OR “pathway*” OR “referral*” OR “utilisation” OR “utilization” OR “use” OR “uptake” ) |  |
|  | S3 | TS= ( “barrier*” OR “determinant*” OR “difference*” OR “disadvantage*” OR “discriminat*” OR “disparit*” OR “equal*” OR “equit*” OR “facilitator*” OR “inequal*” OR “inequit*” OR “intersectional*” OR “minorit*” OR “unequal” OR “unfair” OR “variation*” ) |  |
|  | S4 | TS= ( “united kingdom” OR “uk” OR “england” OR “wales” OR “scotland” OR “northern ireland” OR “national health service” OR “nhs” OR “london” ) |  |
|  | S1 AND S2 AND S3 AND S4 | Limited to English language  Limited to 2014+ |  |
