# Supplementary material for: Understanding inequalities in access to adult mental health services in the UK: a systematic mapping review
Source: BMC Health Serv Res. 2023 Sep 29;23:1042. doi: 10.1186/s12913-023-10030-8 (PMC10542667; doi:10.1186/s12913-023-10030-8)
Supplement: Supplementary file 3 — Additional file 3: Table S3. Summary of included studies. [file 12913_2023_10030_MOESM3_ESM.docx]

**Additional file 3**

**Table S3.** Summary of included studies

| **Author** | **Year** | **Study location** | **Study aim** | **Study setting** | **Study type - design** | **Evidence of PPI** | **Used routinely collected data** | **Measuring access – using Levesque framework** | **Main dimensions of inequality studied** | **Ref** |
| --- | --- | --- | --- | --- | --- | --- | --- | --- | --- | --- |
| Fernandez de la Cruz et al. | 2016 | London, England | to explore illness perceptions, help-seeking attitudes, knowledge about the disorder, and causal attributions in individuals from four different ethnic groups | Secondary care | Quantitative - Questionnaire/survey | No | No | Perception of needs and desire for care | Race, ethnicity, culture, and language | (1) |
| Liberati et al. | 2022 | England | to use the candidacy construct to enable a theoretically informed examination of access to secondary MH services during COVID-19 | Secondary care | Qualitative - Interview | Yes | No | Perception of needs and desire for care | Multiple/exploratory | (2) |
| Adams et al. | 2022 | North East England | to understand the experiences of people who experienced homelessness during the COVID-19 with accessing MH support | Other | Qualitative - Interview | Yes | No | Healthcare seeking | Multiple/exploratory | (3) |
| Arday | 2018 | UK | to examine the impact of negotiating racial inequality and discrimination at university and the impact on MH | Other | Qualitative –  Questionnaire / survey | No | No | Healthcare seeking | Race, ethnicity, culture, and language | (4) |
| Bailey & Tribe | 2021 | UK | to explore experiences that underlie help-seeking among UK resident older Black Caribbean adults, and to explore barriers experienced by participants in seeking help from MH services | Other | Qualitative - Interview | No | No | Healthcare seeking | Race, ethnicity, culture, and language | (5) |
| Bellesi et al. | 2020 | London, England | to understand why individuals of Black Caribbean origin benefit less from therapy, and what changes could be made to make service provision more culturally relevant | IAPT | Qualitative - Focus group | No | No | Healthcare seeking | Race, ethnicity, culture, and language | (6) |
| Berry et al. | 2020 | North West England | to identify the barriers to accessing psychological therapies for severe MH difficulties in later life | Multiple | Qualitative - Interview | Yes | No | Healthcare seeking | Age | (7) |
| Brooks et al. | 2017 | London, England | to explore patients' views about accessing and experiencing treatment | Other | Qualitative - Interview | No | No | Healthcare seeking | Occupation | (8) |
| Brown et al. | 2014 | London, England | to contrast patterns of informal and formal help-seeking using data from a community psychiatric morbidity survey | Other | Quantitative - Questionnaire/survey | No | No | Healthcare seeking | Multiple/exploratory | (9) |
| Bryant et al. | 2022 | Midlands, England | to examine which variables best predict help-seeking from informal and formal sources of MH support | Other | Quantitative - Questionnaire/survey | No | No | Healthcare seeking | Education | (10) |
| Bu et al. | 2021 | UK | to examine how engagement with both formal MH support and informal MH support during COVID-19 varied amongst individuals | Other | Quantitative - Questionnaire/survey | No | No | Healthcare seeking | Multiple/exploratory | (11) |
| Chui et al. | 2021 | London, England | to identify inequalities in referral source by age, ethnicity, migration status and gender, and to examine differences in referral destination by age, ethnicity, migration status and gender | Secondary care | Quantitative - Observational | No | Yes | Healthcare seeking | Multiple/exploratory | (12) |
| Daniels et al. | 2021 | UK | to understand the experiences of psychologically distressed doctors working on the frontline during COVID-19 | Other | Qualitative - Interview | Yes | No | Healthcare seeking | Occupation | (13) |
| Dockery et al. | 2015 | London, England | to establish the frequency of stigma and non-stigma related treatment barriers to MH care, and investigate demographic and clinical variables associated with stigma-related MH care barriers | Secondary care | Mixed - Multiple | No | No | Healthcare seeking | Multiple/exploratory | (14) |
| Ennis et al. | 2019 | Northern Ireland | to assess treatment access, intentions to seek help, and perceived barriers to help-seeking, considering gender | Other | Quantitative - Questionnaire/survey | No | No | Healthcare seeking | Gender | (15) |
| Fertout et al. | 2015 | UK | to carry out a PCA of a stigma/BTC scale and assess the association of the derived components with MH symptoms and help-seeking activity | Other | Quantitative - Questionnaire/survey | No | No | Healthcare seeking | Occupation | (16) |
| Foy et al. | 2019 | England | to identify LGBQ+ adults' experiences of accessing and receiving psychological interventions from IAPT services | IAPT | Mixed - Questionnaire/survey | No | No | Healthcare seeking | Sexual orientation | (17) |
| Gillard et al. | 2021 | UK | to explore the experiences of a range of people with pre-existing MH problems during COVID-19 | Other | Qualitative - Interview | Yes | No | Healthcare seeking | Multiple/exploratory | (18) |
| Gondek & Kirkbride | 2018 | UK | to assess the association between predictors (predisposing, enabling, need) and past help-seeking behaviours and intentions of future help-seeking | Other | Quantitative - Questionnaire/survey | No | No | Healthcare seeking | Race, ethnicity, culture, and language | (19) |
| Harrop et al. | 2021 | UK | to investigate bereavement support needs and experiences in the UK during COVID-19 | Other | Mixed - Questionnaire/survey | No | No | Healthcare seeking | Multiple/exploratory | (20) |
| Islam et al. | 2015 | Birmingham, England | to examine cultural appropriateness, accessibility, and acceptability of EIP services in Birmingham for BME patients | Secondary care | Qualitative - Focus group | Yes | No | Healthcare seeking | Race, ethnicity, culture, and language | (21) |
| Kanakam | 2022 | London, England | to understand therapists' perspectives on how ethnic minority females diagnosed with ED access specialist services | Secondary care | Qualitative - Interview | No | No | Healthcare seeking | Race, ethnicity, culture, and language | (22) |
| Kennedy et al. | 2016 | UK | to evaluate feasibility of self-referral to MH services within a military environment | Tertiary care | Quantitative - Observational | No | Yes | Healthcare seeking | Occupation | (23) |
| Khanom et al. | 2021 | Wales | to identify barriers and facilitators to access to healthcare for asylum seekers and refugees in Wales | Other | Qualitative - Focus group | Yes | No | Healthcare seeking | Refugees and asylum seekers | (24) |
| McGrath et al. | 2020 | South England | to investigate barriers to accessing psychological treatment for male young offenders detained in UK prison | Other | Quantitative - Multiple | Yes | Yes | Healthcare seeking | Contact with criminal justice system | (25) |
| Mellotte et al. | 2017 | England | to understand the barriers and enablers to seeking professional help for veterans | Tertiary care | Mixed - Interview | No | No | Healthcare seeking | Occupation | (26) |
| Memon et al. | 2016 | South East England | to determine perceived barriers to accessing MH services among people from BME backgrounds | Other | Qualitative - Focus group | No | No | Healthcare seeking | Race, ethnicity, culture, and language | (27) |
| Millett et al. | 2018 | England | to investigate how women view IAPT support for perinatal MH | IAPT | Qualitative - Interview | No | No | Healthcare seeking | Pregnancy and maternity | (28) |
| Moller et al. | 2016 | North England | to explore attitudes and beliefs that second-generation South Asian women living in Britain hold about counselling, and how these beliefs impact on help-seeking for psychological distress | Other | Qualitative - Questionnaire/survey | No | No | Healthcare seeking | Race, ethnicity, culture, and language | (29) |
| Morris et al. | 2022 | UK | to investigate the experiences of sexual minorities who attempted to access and receive IAPT services for mild to moderate psychological problems | IAPT | Qualitative - Interview | No | No | Healthcare seeking | Sexual orientation | (30) |
| Murphy et al. | 2014 | South East England | to examine the factors which facilitate UK military personnel with PTSD to engage in help-seeking behaviours | Tertiary care | Qualitative - Interview | Yes | No | Healthcare seeking | Occupation | (31) |
| Ogueji et al. | 2022 | UK | to explore factors restricting professional help-seeking practices among Black family members in low and middle socioeconomic groups in the UK and Nigeria | Other | Mixed - Questionnaire/survey | Yes | No | Healthcare seeking | Race, ethnicity, culture, and language | (32) |
| Paudyal et al. | 2021 | South East England | to explore the mental wellbeing of Syrian refugees and their coping mechanisms and pathways towards community integration | Other | Qualitative - Interview | No | No | Healthcare seeking | Refugees and asylum seekers | (33) |
| Pilav et al. | 2022 | London, England | to explore the multi-level barriers Black, Asian, and minority ethnic women experience when accessing MH services in the perinatal period | Secondary care | Qualitative - Interview | No | No | Healthcare seeking | Race, ethnicity, culture, and language | (34) |
| Pilav et al. | 2022 | London, England | to explore minority ethnic women's experience of perinatal MH services during COVID-19 | Secondary care | Qualitative - Interview | No | No | Healthcare seeking | Race, ethnicity, culture, and language | (35) |
| Rabiee & Smith | 2014 | Birmingham, England | to examine understanding of MH and the extent to which statutory and voluntary MH services in Birmingham are meeting the needs of a range of Black African and African Caribbean communities | Other | Qualitative - Multiple | No | No | Healthcare seeking | Race, ethnicity, culture, and language | (36) |
| Rafferty et al. | 2019 | UK | to explore the barriers and facilitators to accessing professional MH support for veterans | Other | Qualitative - Interview | Yes | No | Healthcare seeking | Occupation | (37) |
| Reader et al. | 2017 | North Wales | to describe the experiences of Deaf people who have used MH services in North Wales | Other | Qualitative - Interview | No | No | Healthcare seeking | Disability | (38) |
| Sagar-Ouriaghli et al. | 2020 | London, England | to identify potential approaches that would be relevant to improving MH help-seeking in male students | Other | Qualitative - Focus group | Yes | No | Healthcare seeking | Gender | (39) |
| Salaheddin & Mason | 2016 | UK | to investigate why young adults may choose not to seek any support for an emotional or MH difficulty | Other | Mixed - Questionnaire/survey | Yes | No | Healthcare seeking | Age | (40) |
| Sancho & Larkin | 2020 | London, England | to understand barriers and facilitators to accessing MH services in the UK for Afro-Caribbean undergraduate students | Other | Qualitative - Focus group | No | No | Healthcare seeking | Race, ethnicity, culture, and language | (41) |
| Shah et al. | 2022 | England & Wales | to explore whether and how participants' day-to-day experiences and MH difficulties changed or stayed the same for participants since their first interview during COVID-19 | Other | Qualitative - Interview | Yes | No | Healthcare seeking | Multiple/exploratory | (42) |
| Simkhada et al. | 2021 | South England | to explore the relationship between culture and access to MH services among Nepali and Iranian migrants in the UK | Other | Qualitative - Interview | No | No | Healthcare seeking | Race, ethnicity, culture, and language | (43) |
| Spiers et al. | 2017 | England | to establish what might help or hinder GPs experiencing mental distress as they consider seeking help for their symptoms | Other | Qualitative - Interview | No | No | Healthcare seeking | Occupation | (44) |
| Stevelink et al. | 2019 | UK | to examine and describe sources of support, prevalence, and associates of help-seeking among UK serving and ex-serving personnel | Other | Quantitative - Interview | No | No | Healthcare seeking | Occupation | (45) |
| Thompson et al. | 2022 | UK | to explore the MH support needs of Gypsy, Roma, and Traveller people within the British Isles | Other | Qualitative - Interview | No | No | Healthcare seeking | Race, ethnicity, culture, and language | (46) |
| Watson & Soltani | 2019 | North England | to investigate ethnic minority women's experiences and opinions of perinatal MH problems and the provision support services | Other | Mixed - Questionnaire/survey | Yes | No | Healthcare seeking | Pregnancy and maternity | (47) |
| Williamson et al. | 2019 | UK | to examine whether perceptions of stigma and barriers to care differed in a UK military sample between those with and without a current likely MH diagnosis | Other | Quantitative - Interview | No | No | Healthcare seeking | Occupation | (48) |
| Williamson et al. | 2021 | UK | to examine how UK military veterans with complex PTSD engage with psychological services | Other | Qualitative - Interview | No | No | Healthcare seeking | Occupation | (49) |
| Yeung et al. | 2017 | England | to examine how Chinese populations make sense of mental distress, and how this influences their pathways to MH care | Other | Qualitative - Interview | No | No | Healthcare seeking | Race, ethnicity, culture, and language | (50) |
| Butterworth et al. | 2017 | West Midlands, England | to understand weaknesses in the current MH and social care pathway to inform development of transition support services | Other | Qualitative - Interview | Yes | No | Healthcare reaching | Age | (51) |
| Carroll et al. | 2021 | London, England | to explore therapists' perceptions of barriers and facilitators to uptake and engagement with therapy in long-term conditions | Other | Qualitative - Interview | No | No | Healthcare reaching | Disability | (52) |
| Chinn & Abraham | 2016 | England | to examine how the legitimacy of claims by people with intellectual disabilities to use IAPT services is impeded or facilitated | IAPT | Mixed - Multiple | Yes | No | Healthcare reaching | Disability | (53) |
| Gregson et al. | 2022 | UK | to understand the experiences of psychologists delivering psychological services to people with learning disabilities during COVID-19 | Secondary care | Qualitative - Interview | Yes | No | Healthcare reaching | Disability | (54) |
| Plugge et al. | 2014 | Berkshire, England | to explore issues around health and access to health services for those on probation | Other | Qualitative - Focus group | No | No | Healthcare reaching | Contact with criminal justice system | (55) |
| Potter et al. | 2022 | UK | to synthesise experiences of professionals who work with street sex workers and what healthcare services are available, and how accessible and effective they are | Other | Mixed - Questionnaire/survey | Yes | No | Healthcare reaching | Trafficked and street sex workers | (56) |
| Sakellariou & Rotarou | 2017 | UK | to investigate differences in access to healthcare between people with and without disabilities in the UK | Other | Quantitative - Questionnaire/survey | No | No | Healthcare reaching | Disability | (57) |
| van der Kamp | 2018 | Scotland | to describe the barriers and facilitators to an effective transition from CAMHS to AMHS | Secondary care | Qualitative - Interview | No | No | Healthcare reaching | Age | (58) |
| Watson & Daley | 2015 | London, England | to determine the incidence of the use of section 135 of the MHA in a London borough and describe the main features of the population subject in that section | Secondary care | Quantitative - Observational | No | Yes | Healthcare reaching | Multiple/exploratory | (59) |
| Williamson et al. | 2019 | England & Scotland | to investigate barriers that hinder healthcare providers from identifying, providing care and making necessary referrals for trafficked people in the UK | Multiple | Qualitative - Interview | No | No | Healthcare reaching | Trafficked and street sex workers | (60) |
| Ajnakina et al. | 2017 | London, England | to investigate clinical and social outcomes in Black African and Caribbean ethnic groups compared with White British MH patients | Secondary care | Quantitative - Multiple | No | Yes | Healthcare utilisation | Race, ethnicity, culture, and language | (61) |
| Anselmi et al. | 2020 | England | to produce a revised formula to inform CCG allocations for secondary MH care provision | Multiple | Quantitative - Observational | No | Yes | Healthcare utilisation | Multiple/exploratory | (62) |
| Bansal et al. | 2014 | Scotland | to use linked data to investigate ethnic variations in psychiatric hospitalisations and compulsory treatment under MHA in Scotland | Secondary care | Quantitative - Observational | No | Yes | Healthcare utilisation | Race, ethnicity, culture, and language | (63) |
| Bebbington et al. | 2017 | London, England | to report the frequency of psychiatric morbidity by sex and sentencing status | Other | Quantitative - Interview | No | No | Healthcare utilisation | Contact with criminal justice system | (64) |
| Bhavsar et al. | 2021 | London, England | to assess ethnic and migration-related differences in IAPT-based psychological treatment use | Other | Quantitative - Multiple | No | Yes | Healthcare utilisation | Race, ethnicity, culture, and language | (65) |
| Brown et al. | 2014 | London, England | to assess whether an IAPT service is delivering an equitable service in a London borough, by comparing socio-demographic and socio-economic characteristics of patients using IAPT services with those who had MH needs identified in a community psychiatric study | IAPT | Quantitative - Multiple | No | Yes | Healthcare utilisation | Multiple/exploratory | (66) |
| Butler et al. | 2021 | London, England | to characterise referrals made to inpatient liaison psychiatry service before and during COVID-19 | Secondary care | Quantitative - Observational | No | Yes | Healthcare utilisation | Multiple/exploratory | (67) |
| Byrne et al. | 2019 | London, England | to understand if when offered treatment, do Black and ethnic minority service users at risk for psychosis engage in the same way as White British service users | Secondary care | Quantitative - Observational | No | Yes | Healthcare utilisation | Race, ethnicity, culture, and language | (68) |
| Carr et al. | 2016 | England | to examine primary care clinical management following an episode of self-harm using data from GPs | Primary care | Quantitative - Observational | No | Yes | Healthcare utilisation | Multiple/exploratory | (69) |
| Carruthers & Oakeshott | 2019 | South London, England | to understand how often refugees and asylum seekers consult primary care doctors, what they consult primary care doctors about, and if secondary care referrals are made | Primary care | Quantitative - Observational | No | Yes | Healthcare utilisation | Refugees and asylum seekers | (70) |
| Chaplin et al. | 2015 | England & Wales | to calculate relative access of older adults in comparison to adults of working age to psychological services, and assess treatment experiences and outcomes | IAPT | Mixed - Observational | No | Yes | Healthcare utilisation | Age | (71) |
| Chen et al. | 2020 | Cambridge & Peterborough, England | to investigate the impact of lockdown on referrals to secondary care MH clinical services, and perform sub-group analyses for vulnerable groups | Secondary care | Quantitative - Observational | No | Yes | Healthcare utilisation | Multiple/exploratory | (72) |
| Clement et al. | 2015 | England | to test the hypothesis that experienced MH-related discrimination is associated with low engagement among adults receiving care from CMHTs | Secondary care | Quantitative - Interview | No | Yes | Healthcare utilisation | Multiple/exploratory | (73) |
| Colling et al. | 2017 | London, England | to examine whether demographic characteristics differentially predicted receipt of CBTp | Secondary care | Quantitative - Observational | Yes | Yes | Healthcare utilisation | Multiple/exploratory | (74) |
| Cullen et al. | 2018 | London, England | to determine the demographic, clinical and behavioural predictors of PICU and seclusion | Secondary care | Quantitative - Observational | No | Yes | Healthcare utilisation | Multiple/exploratory | (75) |
| Dagnan et al. | 2022 | England | to present national IAPT data to explore outcomes for people with learning disabilities compared with people without learning disabilities | IAPT | Quantitative - Observational | No | Yes | Healthcare utilisation | Disability | (76) |
| Das-Munshi et al. | 2018 | England & Wales | to assess access to evidence-based treatments for psychosis amongst main ethnic minority groups | Secondary care | Quantitative - Multiple | No | Yes | Healthcare utilisation | Race, ethnicity, culture, and language | (77) |
| Day et al. | 2021 | London, Oxford, & Newcastle, England | to explore the extent and nature of treatment gaps experienced by a sample of patients with established treatment-resistant depression | Multiple | Quantitative - Observational | No | No | Healthcare utilisation | Multiple/exploratory | (78) |
| Delgadillo et al. | 2016 | England | to examine the relationships between socioeconomic deprivation with referrals, access to therapy, and clinical outcomes in IAPT services in England | IAPT | Quantitative - Observational | No | Yes | Healthcare utilisation | Socio-economic status | (79) |
| Delgadillo et al. | 2018 | England | to gather workforce size estimates for a representative sample of IAPT services and investigate associations between socio-economic deprivation, workforce size, and treatment access | IAPT | Mixed - Questionnaire/survey | No | Yes | Healthcare utilisation | Multiple/exploratory | (80) |
| Di Bona et al. | 2014 | Doncaster & Newham, England | to analyse socio-demographic and clinical data on patients referred to IAPT services by their GP and whether or not they accessed IAPT services | IAPT | Quantitative - Multiple | No | Yes | Healthcare utilisation | Multiple/exploratory | (81) |
| Domoney et al. | 2015 | London, England | to understand how people are identified as trafficked within MH services and challenges associated with responding to trafficked people's MH needs | Secondary care | Qualitative - Observational | No | Yes | Healthcare utilisation | Trafficked and street sex workers | (82) |
| Dorrington et al. | 2021 | London, England | to explore the extent to which people receiving fit notes access MH treatment across primary and secondary care, and demographic variations | Primary care | Quantitative - Observational | No | Yes | Healthcare utilisation | Multiple/exploratory | (83) |
| Ellis et al. | 2015 | UK | to gain a better understanding of experiences of trans people as a whole and evaluate MH services and GIC services for trans clients | Other | Mixed - Questionnaire/survey | Yes | No | Healthcare utilisation | Gender | (84) |
| Fernandez de la Cruz et al. | 2015 | London, England | to explore whether individuals with OCD from ethnic minorities are under-represented in secondary and tertiary services within a large MH trust in South London | Secondary care | Quantitative - Observational | No | Yes | Healthcare utilisation | Race, ethnicity, culture, and language | (85) |
| Firth et al. | 2020 | North England | to investigate impact of socio-demographic similarity on the probability of an adequate dose of a psychoeducational group intervention | IAPT | Quantitative - Observational | No | Yes | Healthcare utilisation | Multiple/exploratory | (86) |
| Forrester et al. | 2017 | London, England | to describe demographic and clinical characteristics of the first consecutive cohort of referrals over an 18-month period | Tertiary care | Quantitative - Observational | No | Yes | Healthcare utilisation | Contact with criminal justice system | (87) |
| Gajwani et al. | 2016 | Birmingham, England | to examine ethnic differences in patients assessed for detention and explore the effect of ethnicity after controlling for confounders | Secondary care | Quantitative - Observational | No | Yes | Healthcare utilisation | Race, ethnicity, culture, and language | (88) |
| Gazard et al. | 2018 | London, England | to investigate differences in health service use and examine the role of discrimination experiences | Other | Quantitative - Questionnaire/survey | No | No | Healthcare utilisation | Multiple/exploratory | (89) |
| Giebel et al. | 2014 | North West England | to investigate the demographic and clinical characteristics of subgroups of veterans attending IAPT services | IAPT | Quantitative - Observational | No | Yes | Healthcare utilisation | Occupation | (90) |
| Giebel et al. | 2020 | North West England | to explore whether access to MH treatments differed by socio-economic status | Other | Quantitative - Questionnaire/survey | Yes | No | Healthcare utilisation | Socio-economic status | (91) |
| Gnan et al. | 2019 | UK | to investigate general and LGBTQ-specific factors associated with having a current MH problem, use of MH services, suicide risk and self-harm in university students | Other | Quantitative - Questionnaire/survey | No | No | Healthcare utilisation | Sexual orientation | (92) |
| Godier-McBard et al. | 2022 | UK | to provide a preliminary investigation of gender differences in engagement and barriers to MH care in a sample of UK veterans | Other | Mixed - Questionnaire/survey | No | No | Healthcare utilisation | Gender | (93) |
| Harwood et al. | 2021 | London, England | to examine variation by ethnicity in source of referral, receipt of an initial assessment, and receipt of at least one treatment session within an IAPT service | IAPT | Quantitative - Observational | No | Yes | Healthcare utilisation | Race, ethnicity, culture, and language | (94) |
| Holman | 2014 | England | to understand the underuse of talking treatments by working class people | IAPT | Mixed - Multiple | No | No | Healthcare utilisation | Socio-economic status | (95) |
| Hopkin et al. | 2020 | London, England | to investigate differences in characteristics between homeless and non-homeless people within a population of people referred to MH services in police custody | Tertiary care | Quantitative - Observational | No | Yes | Healthcare utilisation | Place of residence | (96) |
| Jakobowitz et al. | 2017 | London, England | to quantify overall levels of the need for MH care and treatment in prisoners, and assess how far these needs were met by the various MH facilities in prison | Other | Quantitative - Interview | No | No | Healthcare utilisation | Contact with criminal justice system | (97) |
| Jankovic et al. | 2020 | England | to explore access rates to community MH services, rates of inpatient psychiatric hospital admissions and rates of involuntary inpatient psychiatric hospital admissions, and explore whether a higher density of ethnic minority populations is linked to lower access rates | Secondary care | Quantitative - Observational | No | Yes | Healthcare utilisation | Pregnancy and maternity | (98) |
| Kapadia et al. | 2018 | England | to investigate association between ethnic group and MH service usage for women in England | Other | Quantitative - Questionnaire/survey | No | No | Healthcare utilisation | Race, ethnicity, culture, and language | (99) |
| Kirkbride et al. | 2017 | East England | to estimate waiting times to EIP services in a large, representative epidemiological cohort in England | Secondary care | Quantitative - Observational | No | Yes | Healthcare utilisation | Multiple/exploratory | (100) |
| Kothari et al. | 2022 | London, England | to evaluate impact of integrated MH and substance misuse service within a prison setting | Tertiary care | Quantitative - Observational | No | Yes | Healthcare utilisation | Contact with criminal justice system | (101) |
| Lappin et al. | 2016 | London & Nottingham, England | to test the hypothesis that those who develop psychosis at a younger age have worse outcomes than those who develop psychosis at an older age | Secondary care | Quantitative - Multiple | No | Yes | Healthcare utilisation | Age | (102) |
| Lawrence et al. | 2021 | London & Nottingham, England | to investigate the long-term experience of living with psychosis and navigating MH services within different ethnic groups | Secondary care | Qualitative - Interview | No | No | Healthcare utilisation | Race, ethnicity, culture, and language | (103) |
| Lawrence et al. | 2021 | London & Nottingham, England | to explore the journey through MH services from the perspective of individuals from Black Caribbean and majority White British population to help understand variation in the use of MH services | Secondary care | Qualitative - Interview | No | No | Healthcare utilisation | Race, ethnicity, culture, and language | (104) |
| Leavey et al. | 2019 | Northern Ireland | to examine the pathways and determinants of transition, including the role of social class | Secondary care | Quantitative - Observational | No | Yes | Healthcare utilisation | Age | (105) |
| Livanou et al. | 2020 | England | to examine clinical characteristics, transition pathways, and psychosocial indicators of transition outcomes for young people in forensic secure services discharged to adult services | Tertiary care | Quantitative - Observational | No | Yes | Healthcare utilisation | Age | (106) |
| Livanou et al. | 2020 | England | to map a national sample of young people across all adolescent forensic medium secure units to understand discharge placements and transition timelines | Tertiary care | Mixed - Questionnaire/survey | No | Yes | Healthcare utilisation | Age | (107) |
| Livanou et al. | 2021 | England | to explore the views and experiences of key professionals involved in the transition process from adolescent medium secure units to adult secure and community services in England | Tertiary care | Qualitative - Interview | No | No | Healthcare utilisation | Age | (108) |
| Maconick et al. | 2021 | England | to investigate association between area level factors and number of people in contact with secondary MH services by CCG in England | Other | Quantitative - Observational | No | Yes | Healthcare utilisation | Multiple/exploratory | (109) |
| Majid et al. | 2016 | Birmingham, England | to explore repetition, service provision and service engagement following presentation of young people to emergency services with self-harm | Secondary care | Quantitative - Observational | No | Yes | Healthcare utilisation | Age | (110) |
| Manescu et al. | 2020 | England | to examine the relationship between attitudes to mental illness, symptoms of CMD, seeking help, and receiving medication | Other | Quantitative - Questionnaire/survey | No | No | Healthcare utilisation | Multiple/exploratory | (111) |
| Mankiewicz et al. | 2021 | London, England | to investigate equality of access to family intervention for psychosis, and subsequent treatment uptake and engagement | Secondary care | Quantitative - Observational | No | Yes | Healthcare utilisation | Multiple/exploratory | (112) |
| Mann et al. | 2014 | London, England | to explore ethnic differences in compulsory detention and hospitalisation rates for EIS patients | Secondary care | Quantitative - Observational | No | Yes | Healthcare utilisation | Race, ethnicity, culture, and language | (113) |
| Mansour et al. | 2020 | London, England | to compare symptoms and types of treatment between ethnic groups in patients with late-life depression | Secondary care | Quantitative - Observational | No | Yes | Healthcare utilisation | Race, ethnicity, culture, and language | (114) |
| Mark et al. | 2020 | London, England | to investigate the utility and feasibility of identifying veterans accessing secondary MH services using EHRs | Secondary care | Quantitative - Observational | No | Yes | Healthcare utilisation | Occupation | (115) |
| Matthew Prina et al. | 2014 | East England | to explore differences in referrals and waiting time to access IAPT services between younger and older adults | IAPT | Quantitative - Observational | No | Yes | Healthcare utilisation | Age | (116) |
| McKenzie et al. | 2019 | London, England | to present findings for treatment needs and how far these needs are met for Black and minority ethnic prisoners compared to White prisoners | Other | Quantitative - Interview | No | No | Healthcare utilisation | Contact with criminal justice system | (117) |
| McNamara et al. | 2017 | England | to explore issues associated with referrals to AMHS from CAMHS from a social identity perspective | Secondary care | Qualitative - Interview | No | No | Healthcare utilisation | Age | (118) |
| Meddings et al. | 2019 | Sussex, England | to explore if different groups of people access Recovery College equitably, and if students are representative of the local population and those using MH services | Secondary care | Quantitative - Observational | No | Yes | Healthcare utilisation | Multiple/exploratory | (119) |
| Mercer et al. | 2019 | London, England | to explore differences in access to, and outcomes of, psychological therapy for different ethnic groups across secondary MH care | Secondary care | Quantitative - Observational | No | Yes | Healthcare utilisation | Race, ethnicity, culture, and language | (120) |
| Mirza et al. | 2019 | North England | to examine cultural differences in causal beliefs and stigma toward MH | Other | Quantitative - Questionnaire/survey | No | No | Healthcare utilisation | Race, ethnicity, culture, and language | (121) |
| Moore et al. | 2019 | England | to explore the relationship between ethnicity, migration and MH indicators among mothers participating in a large nationally representative cohort study | Other | Quantitative - Interview | No | No | Healthcare utilisation | Race, ethnicity, culture, and language | (122) |
| Morgan | 2014 | London, England | to provide information regarding the extent to which the process of clustering using the MH clustering tool captures the complexity of patient need across different geographical areas | Secondary care | Quantitative - Observational | No | Yes | Healthcare utilisation | Multiple/exploratory | (123) |
| Morgan et al. | 2017 | London & Nottingham, England | to investigate patterns and determinants of long-term course and outcome of psychosis by ethnic group following a first episode | Secondary care | Quantitative - Observational | No | Yes | Healthcare utilisation | Race, ethnicity, culture, and language | (124) |
| Nicholson & Hotchin | 2015 | Glasgow & Clyde, Scotland | to investigate the relationship between area deprivation and contact with ID psychiatry | Secondary care | Quantitative - Observational | No | Yes | Healthcare utilisation | Socio-economic status | (125) |
| Nilforooshan et al. | 2017 | London, England | to investigate the differences in service utilisation and costs between working age adults and older adults across five mental health healthcare providers in and around London | Secondary care | Quantitative - Observational | No | Yes | Healthcare utilisation | Age | (126) |
| Oates & Firth | 2020 | Derby, England | to evaluate the extent to which IMD predicted access to treatment, attendance, treatment completion and clinical outcomes in a British health psychology clinic | Secondary care | Quantitative - Observational | No | Yes | Healthcare utilisation | Socio-economic status | (127) |
| Oduola et al. | 2019 | London, England | to investigate whether disparities in pathways to care for those from minority ethnic groups continue | Secondary care | Quantitative - Observational | No | Yes | Healthcare utilisation | Multiple/exploratory | (128) |
| Pettit et al. | 2017 | South West England | to accurately estimate differences in referral and access rates to the IAPT services and compare the pathway through treatment across age bands | IAPT | Quantitative - Questionnaire/survey | No | Yes | Healthcare utilisation | Age | (129) |
| Prady et al. | 2016 | Bradford, England | to examine the quantity and types of treatment offered to women with CMD before, during and up to one year postnatally, and assess psychological treatment variation by ethnic group | Secondary care | Quantitative - Multiple | No | Yes | Healthcare utilisation | Pregnancy and maternity | (130) |
| Reichert & Jacobs | 2018 | England | to investigate inequalities in duration of untreated psychosis associated with socioeconomic deprivation in England | Secondary care | Quantitative - Observational | No | Yes | Healthcare utilisation | Socio-economic status | (131) |
| Rhead et al. | 2022 | England | to examine MH service use and treatment at the intersections of multiple advantaged and disadvantaged social statuses | Other | Quantitative - Questionnaire/survey | No | No | Healthcare utilisation | Multiple/exploratory | (132) |
| Ride et al. | 2020 | England | to estimate annual healthcare costs for people with SMI in England across primary and secondary care settings | Multiple | Quantitative - Observational | No | Yes | Healthcare utilisation | Multiple/exploratory | (133) |
| Saini et al. | 2021 | Liverpool, England | to compare help-seeking among younger and older men who attended a therapeutic centre for men in suicidal crisis | Other | Quantitative - Observational | No | Yes | Healthcare utilisation | Age | (134) |
| Singh et al. | 2015 | Birmingham, England | to understand if ethnic groups significantly differ in culturally mediated illness attributions during FEP, and if ethnic groups significantly differ in their pathways to care during FEP | Secondary care | Quantitative - Multiple | No | Yes | Healthcare utilisation | Race, ethnicity, culture, and language | (135) |
| Sizmur & McCullough | 2016 | England | to analyse survey variables describing treatment offered to respondents for evidence of differential access to services associated with ethnicity | Secondary care | Quantitative - Questionnaire/survey | No | No | Healthcare utilisation | Race, ethnicity, culture, and language | (136) |
| Smyth et al. | 2022 | London, England | to explore the association between sociodemographic and socioeconomic indicators and the use of psychological treatment services | IAPT | Quantitative - Observational | No | Yes | Healthcare utilisation | Multiple/exploratory | (137) |
| Steeg et al. | 2022 | UK | to examine the impact of COVID-19 on clinical management within 3 months of a self-harm episode | Primary care | Quantitative - Observational | Yes | Yes | Healthcare utilisation | Multiple/exploratory | (138) |
| Thomas et al. | 2020 | South West England | to examine IAPT referrals made by GPs and how these referrals are perceived and acted on by patients from low-income backgrounds | Other | Qualitative - Interview | Yes | No | Healthcare utilisation | Socio-economic status | (139) |
| Tseliou et al. | 2017 | London, England | to explore gender differences for first-presentation psychosis patients at the time of referral to inner-city EIS and one year later | Secondary care | Quantitative - Observational | No | Yes | Healthcare utilisation | Gender | (140) |
| Tucker et al. | 2015 | England | to identify the characteristics of community dwelling older people supported by CMHTs in England | Secondary care | Quantitative - Observational | No | Yes | Healthcare utilisation | Age | (141) |
| Tyler et al. | 2019 | South England | to measure the prevalence and comorbidity of MH needs across a representative sample of both men and women across 13 prisons | Other | Quantitative - Questionnaire/survey | No | No | Healthcare utilisation | Contact with criminal justice system | (142) |
| Valmaggia et al. | 2015 | London, England | to compare sociodemographic features, DUP, hospital admission, and frequency of compulsory treatment in the first year after the onset of psychosis in patients who present to services with patients who did not present | Secondary care | Quantitative - Observational | No | Yes | Healthcare utilisation | Multiple/exploratory | (143) |
| Volkert et al. | 2018 | London, England | to identify factors associated with service utilisation in the elderly | Other | Quantitative - Interview | No | No | Healthcare utilisation | Age | (144) |
| Walters et al. | 2018 | England | to investigate variation in treatment patterns for depression by age, gender, deprivation, and neighbourhood in primary care treatment for depression in older adults in England | Primary care | Quantitative - Observational | No | Yes | Healthcare utilisation | Age | (145) |
| Watson et al. | 2021 | London, England | to assess whether technology, accessibility, and demographic factors influence remote therapy uptake among individuals with psychosis | Secondary care | Quantitative - Questionnaire/survey | No | Yes | Healthcare utilisation | Multiple/exploratory | (146) |
| Weich et al. | 2017 | England | to describe and model spatial variation in compulsory admissions in England using national patient-level data | Secondary care | Quantitative - Observational | No | Yes | Healthcare utilisation | Multiple/exploratory | (147) |
| White et al. | 2014 | England | to explore socio-economic equity of hospital care utilisation for patients with SMI and how it has changed over time | Secondary care | Quantitative - Observational | No | Yes | Healthcare utilisation | Socio-economic status | (148) |
| Wilberforce et al. | 2015 | England | to determine the extent to which services provided to older people via CMHTs vary in duration, composition, and intensity, and identify to what extent differences are due to case mix | Secondary care | Quantitative - Observational | No | Yes | Healthcare utilisation | Age | (149) |
| Yasmin-Qureshi & Ledwith | 2020 | England | to explore South Asian women's experiences of accessing psychological therapy | IAPT | Qualitative - Interview | No | No | Healthcare utilisation | Race, ethnicity, culture, and language | (150) |
| Liberati et al. | 2021 | England | to report a large interview-based study involving people with direct experience of seeking and providing MH care in England during COVID-19 | Secondary care | Qualitative - Interview | Yes | No | Healthcare consequences | Multiple/exploratory | (151) |
| Wiginton et al. | 2021 | England & Wales | to report the prevalence of unmet peer support, psychological care, management of chronic health condition, and isolation help needs in people living with HIV | Other | Quantitative - Questionnaire/survey | No | No | Healthcare consequences | Disability | (152) |

* AMHS, adult mental health services; BME, Black and minority ethnic; BTC, Barriers To Care; CAMHS, child and adolescent mental health services; CBTp, cognitive behaviour therapy for psychosis; CCG, clinical commissioning group; CMD, common mental disorder; CMHT, community mental health team; COVID-19, coronavirus 19; DUP, duration of untreated psychosis; ED, eating disorder; EHR, electronic health record; EIP, early intervention for psychosis; FEP, first episode psychosis; GIC, gender identity clinic; GP, general practitioner; HIV, human immunodeficiency virus; IAPT, improving access to psychological therapies; ID, intellectual disability; IMD, index of multiple deprivation; LGBQ+, lesbian, gay, bisexual, and queer; LGBTQ+, lesbian, gay, bisexual, transgender, and queer; MH, mental health; MHA, Mental Health Act; OCD, obsessive compulsive disorder; PCA, principal components analysis; PICU, psychiatric intensive care unit; PTSD, post-traumatic stress disorder; SMI, severe mental illness; UK, United Kingdom

**Included studies references**

1. Fernandez de la Cruz L, Kolvenbach S, Vidal-Ribas P, Jassi A, Llorens M, Patel N, et al. Illness perception, help-seeking attitudes, and knowledge related to obsessive-compulsive disorder across different ethnic groups: a community survey. Social Psychiatry and Psychiatric Epidemiology. 2016;51(3):455-64.

2. Liberati E, Richards N, Parker J, Willars J, Scott D, Boydell N, et al. Qualitative study of candidacy and access to secondary mental health services during the COVID-19 pandemic. Social Science & Medicine. 2022;296:114711.

3. Adams EA, Parker J, Jablonski T, Kennedy J, Tasker F, Hunter D, et al. A Qualitative Study Exploring Access to Mental Health and Substance Use Support among Individuals Experiencing Homelessness during COVID-19. International Journal of Environmental Research and Public Health. 2022;19(6):3459.

4. Arday J. Understanding Mental Health: What Are the Issues for Black and Ethnic Minority Students at University? Social Sciences. 2018;7(10):196.

5. Bailey NV, Tribe R. A qualitative study to explore the help-seeking views relating to depression among older Black Caribbean adults living in the UK. International Review of Psychiatry. 2021;33(1-2):113-8.

6. Bellesi G, Jeraj S, Manley J, Tekes S, Basit H, McNulty N, editors. Why do Black Caribbean women benefit less from talking therapies? A pilot study in an inner London IAPT service. Clinical Psychology Forum; 2020.

7. Berry K, Sheardown J, Pabbineedi U, Haddock G, Cross C, Brown LJE. Barriers and facilitators to accessing psychological therapies for severe mental health difficulties in later life. Behavioural and Cognitive Psychotherapy. 2020;48(2):216-28.

8. Brooks SK, Gerada C, Chalder T. The specific needs of doctors with mental health problems: qualitative analysis of doctor-patients' experiences with the Practitioner Health Programme. Journal of Mental Health. 2017;26(2):161-6.

9. Brown JSL, Evans-Lacko S, Aschan L, Henderson MJ, Hatch SL, Hotopf M. Seeking informal and formal help for mental health problems in the community: a secondary analysis from a psychiatric morbidity survey in South London. BMC Psychiatry. 2014;14(1):275.

10. Bryant A, Cook A, Egan H, Wood J, Mantzios M. Help-seeking behaviours for mental health in higher education. Journal of Further and Higher Education. 2022;46(4):522-34.

11. Bu F, Mak HW, Fancourt D. Rates and predictors of uptake of mental health support during the COVID-19 pandemic: an analysis of 26,720 adults in the UK in lockdown. Social Psychiatry and Psychiatric Epidemiology. 2021.

12. Chui Z, Gazard B, MacCrimmon S, Harwood H, Downs J, Bakolis I, et al. Inequalities in referral pathways for young people accessing secondary mental health services in south east London. European Child & Adolescent Psychiatry. 2021;30(7):1113-28.

13. Daniels J, Ingram J, Pease A, Wainwright E, Beckett K, Iyadurai L, et al. The COVID-19 Clinician Cohort (CoCCo) Study: Empirically Grounded Recommendations for Forward-Facing Psychological Care of Frontline Doctors. International Journal of Environmental Research and Public Health. 2021;18(18):18.

14. Dockery L, Jeffery D, Schauman O, Williams P, Farrelly S, Bonnington O, et al. Stigma- and non-stigma-related treatment barriers to mental healthcare reported by service users and caregivers. Psychiatry Research. 2015;228(3):612-9.

15. Ennis E, McLafferty M, Murray E, Lapsley C, Bjourson T, Armour C, et al. Readiness to change and barriers to treatment seeking in college students with a mental disorder. Journal of Affective Disorders. 2019;252:428-34.

16. Fertout M, Jones N, Keeling M, Greenberg N. Mental health stigmatisation in deployed UK Armed Forces: a principal components analysis. Journal of the Royal Army Medical Corps. 2015;161 Suppl 1:i69-i76.

17. Foy AAJ, Morris D, Fernandes V, Rimes KA. LGBQ+ adults’ experiences of Improving Access to Psychological Therapies and primary care counselling services: informing clinical practice and service delivery. Cognitive Behaviour Therapist. 2019;12:e42.

18. Gillard S, Dare C, Hardy J, Nyikavar, a P, Rowan Olive R, et al. Experiences of living with mental health problems during the COVID-19 pandemic in the UK: a coproduced, participatory qualitative interview study. Social Psychiatry and Psychiatric Epidemiology. 2021;56(8):1447-57.

19. Gondek D, Kirkbride JB. Predictors of mental health help-seeking among polish people living the United Kingdom. Bmc Health Services Research. 2018;18:12.

20. Harrop E, Goss S, Farnell D, Longo M, Byrne A, Barawi K, et al. Support needs and barriers to accessing support: Baseline results of a mixed-methods national survey of people bereaved during the COVID-19 pandemic. Palliative Medicine. 2021;35(10):1985-97.

21. Islam Z, Rabiee F, Singh SP. Black and Minority Ethnic Groups' Perception and Experience of Early Intervention in Psychosis Services in the United Kingdom. Journal of Cross-Cultural Psychology. 2015;46(5):737-53.

22. Kanakam N. Therapists' Experiences of Working with Ethnic Minority Females with Eating Disorders: A Qualitative Study. Culture, Medicine, and Psychiatry. 2022;46(2):414-34.

23. Kennedy I, Whybrow D, Jones N, Sharpley J, Greenberg N. A service evaluation of self-referral to military mental health teams. Occupational Medicine. 2016;66(5):394-8.

24. Khanom A, Alanazy W, Couzens L, Evans BA, Fagan L, Fogarty R, et al. Asylum seekers' and refugees' experiences of accessing health care: a qualitative study. BJGP Open. 2021;5(6).

25. McGrath K, Shaw J, Farquharson L. Barriers to accessing psychological treatment for medium to high risk male young offenders. Journal of Forensic Psychiatry & Psychology. 2020;31(4):596-612.

26. Mellotte H, Murphy D, Rafferty L, Greenberg N. Pathways into mental health care for UK veterans: a qualitative study. European Journal of Psychotraumatology. 2017;8(1):11.

27. Memon A, Taylor K, Mohebati LM, Sundin J, Cooper M, Scanlon T, et al. Perceived barriers to accessing mental health services among black and minority ethnic (BME) communities: a qualitative study in Southeast England. BMJ Open. 2016;6(11):9.

28. Millett L, Taylor BL, Howard LM, Bick D, Stanley N, Johnson S. Experiences of Improving Access to Psychological Therapy Services for Perinatal Mental Health Difficulties: a Qualitative Study of Women's and Therapists' Views. Behavioural and Cognitive Psychotherapy. 2018;46(4):421-36.

29. Moller N, Burgess V, Jogiyat Z. Barriers to counselling experienced by British South Asian women: A thematic analysis exploration. Counselling & Psychotherapy Research. 2016;16(3):201-10.

30. Morris DDA, Fernandes V, Rimes KA. Sexual minority service user perspectives on mental health treatment barriers to care and service improvements. International Review of Psychiatry. 2022;34(3-4):230-9.

31. Murphy D, Hunt E, Luzon O, Greenberg N. Exploring positive pathways to care for members of the UK Armed Forces receiving treatment for PTSD: a qualitative study. European Journal of Psychotraumatology. 2014;5:8.

32. Ogueji IA, Okoloba MM. Seeking Professional Help for Mental Illness: A Mixed-Methods Study of Black Family Members in the UK and Nigeria. Psychological Studies. 2022;67(2):164-77.

33. Paudyal P, Tattan M, Cooper MJF. Qualitative study on mental health and well-being of Syrian refugees and their coping mechanisms towards integration in the UK. BMJ Open. 2021;11(8):9.

34. Pilav S, De Backer K, Easter A, Silverio SA, Sundaresh S, Roberts S, et al. A qualitative study of minority ethnic women's experiences of access to and engagement with perinatal mental health care. BMC Pregnancy Childbirth. 2022;22(1):421.

35. Pilav S, Easter A, Silverio SA, De Backer K, Sundaresh S, Roberts S, et al. Experiences of Perinatal Mental Health Care among Minority Ethnic Women during the COVID-19 Pandemic in London: A Qualitative Study. International Journal of Environmental Research and Public Health. 2022;19(4):15.

36. Rabiee F, Smith P. Understanding mental health and experience of accessing services among African and African Caribbean Service users and carers in Birmingham, UK. Diversity and Equality in Health and Care. 2014;11(2):125-34.

37. Rafferty LA, Wessely S, Stevelink SAM, Greenberg N. The journey to professional mental health support: a qualitative exploration of the barriers and facilitators impacting military veterans' engagement with mental health treatment. European Journal of Psychotraumatology. 2019;10(1):14.

38. Reader D, Foulkes H, Robinson C. Investigating barriers to mental health care experienced by the Deaf community in North Wales. Mental Health Nursing. 2017;37(3):14-9.

39. Sagar-Ouriaghli I, Brown JSL, Tailor V, Godfrey E. Engaging male students with mental health support: a qualitative focus group study. BMC Public Health. 2020;20(1):1159.

40. Salaheddin K, Mason B. Identifying barriers to mental health help-seeking among young adults in the UK: a cross-sectional survey. British Journal of General Practice. 2016;66(651):E686-E92.

41. Sancho TN, Larkin M. "We need to slowly break down this barrier": understanding the barriers and facilitators that Afro-Caribbean undergraduates perceive towards accessing mental health services in the UK. Journal of Public Mental Health. 2020;19(1):63-81.

42. Shah P, Hardy J, Birken M, Foye U, Olive RR, Nyikavaranda P, et al. What has changed in the experiences of people with mental health problems during the COVID-19 pandemic: a coproduced, qualitative interview study. Social Psychiatry and Psychiatric Epidemiology. 2022;57(6):1291-303.

43. Simkhada B, Vahdaninia M, van Teijlingen E, Blunt H. Cultural issues on accessing mental health services in Nepali and Iranian migrants communities in the UK. International Journal of Mental Health Nursing. 2021;30(6):1610-9.

44. Spiers J, Buszewicz M, Chew-Graham CA, Gerada C, Kessler D, Leggett N, et al. Barriers, facilitators, and survival strategies for GPs seeking treatment for distress: a qualitative study. British Journal of General Practice. 2017;67(663):e700-e8.

45. Stevelink SAM, Jones N, Jones M, Dyball D, Khera CK, Pernet D, et al. Do serving and ex-serving personnel of the UK armed forces seek help for perceived stress, emotional or mental health problems? European Journal of Psychotraumatology. 2019;10(1):1556552.

46. Thompson RM, Stone BV, Tyson PJ. Mental health support needs within Gypsy, Roma, and Traveller communities: a qualitative study. Mental Health and Social Inclusion. 2022;26(2):144-55.

47. Watson H, Soltani H. Perinatal mental ill health: the experiences of women from ethnic minority groups. British Journal of Midwifery. 2019;27(10):642-8.

48. Williamson V, Greenberg N, Stevelink SAM. Perceived stigma and barriers to care in UK Armed Forces personnel and veterans with and without probable mental disorders. BMC Psychology. 2019;7(1):75.

49. Williamson V, Pearson EJ, Shevlin M, Karatzias T, Macmanus D, Murphy D. Experiences of Veterans with ICD-11 Complex PTSD in Engaging with Services. Journal of Loss & Trauma. 2021;26(2):166-78.

50. Yeung EYW, Irvine F, Ng SM, Tsang KMS. How people from Chinese backgrounds make sense of and respond to the experiences of mental distress: Thematic analysis. Journal of Psychiatric and Mental Health Nursing. 2017;24(8):589-99.

51. Butterworth S, Singh SP, Birchwood M, Islam Z, Munro ER, Vostanis P, et al. Transitioning care-leavers with mental health needs: "they set you up to fail!'. Child and Adolescent Mental Health. 2017;22(3):138-47.

52. Carroll S, Moss-Morris R, Hulme K, Hudson J. Therapists' perceptions of barriers and facilitators to uptake and engagement with therapy in long-term conditions. British Journal of Health Psychology. 2021;26(2):307-24.

53. Chinn D, Abraham E. Using 'candidacy' as a framework for understanding access to mainstream psychological treatment for people with intellectual disabilities and common mental health problems within the English Improving Access to Psychological Therapies service. Journal of Intellectual Disability Research. 2016;60(6):571-82.

54. Gregson N, Randle-Phillips C, Delaney C. Delivering Psychological Services for People with Learning Disabilities during the Covid-19 Pandemic: The Experiences of Psychologists in the UK. Journal of Mental Health Research in Intellectual Disabilities. 2022;15(2):168-96.

55. Plugge E, Pari AAA, Maxwell J, Holl, S. When prison is “easier”: probationers’ perceptions of health and wellbeing. International Journal of Prisoner Health. 2014;10(1):38-46.

56. Potter LC, Horwood J, Feder G. Access to healthcare for street sex workers in the UK: perspectives and best practice guidance from a national cross-sectional survey of frontline workers. BMC Health Services Research. 2022;22(1):178.

57. Sakellariou D, Rotarou ES. Access to healthcare for men and women with disabilities in the UK: secondary analysis of cross-sectional data. BMJ Open. 2017;7(8):e016614.

58. van der Kamp J. The transition between mental health services in Scotland. Mental Health Review Journal. 2018;23(1):12-24.

59. Watson J, Daley S. The use of section 135(1) of the Mental Health Act in a London borough. Mental Health Review Journal. 2015;20(3):133-43.

60. Williamson V, Borschmann R, Zimmerman C, Howard LM, Stanley N, Oram S. Responding to the health needs of trafficked people: A qualitative study of professionals in England and Scotland. Health and Social Care in the Community. 2020;28(1):173-81.

61. Ajnakina O, Lally J, Di Forti M, Kolliakou A, Gardner-Sood P, Lopez-Morinigo J, et al. Patterns of illness and care over the 5 years following onset of psychosis in different ethnic groups; the GAP-5 study. Social Psychiatry and Psychiatric Epidemiology. 2017;52(9):1101-11.

62. Anselmi L, Everton A, Shaw R, Suzuki W, Burrows J, Weir R, et al. Estimating local need for mental healthcare to inform fair resource allocation in the NHS in England: cross-sectional analysis of national administrative data linked at person level. British Journal of Psychiatry. 2020;216(6):338-44.

63. Bansal N, Bhopal R, Netto G, Lyons D, Steiner MFC, Sashidharan SP. Disparate patterns of hospitalisation reflect unmet needs and persistent ethnic inequalities in mental health care: the Scottish health and ethnicity linkage study. Ethnicity & Health. 2014;19(2):217-39.

64. Bebbington P, Jakobowitz S, McKenzie N, Killaspy H, Iveson R, Duffield G, et al. Assessing needs for psychiatric treatment in prisoners: 1. Prevalence of disorder. Social Psychiatry and Psychiatric Epidemiology. 2017;52(2):221-9.

65. Bhavsar V, Jannesari S, McGuire P, MacCabe JH, Das-Munshi J, Bhugra D, et al. The association of migration and ethnicity with use of the Improving Access to Psychological Treatment (IAPT) programme: a general population cohort study. Social Psychiatry and Psychiatric Epidemiology. 2021;56(11):1943-56.

66. Brown JSL, Ferner H, Wingrove J, Aschan L, Hatch SL, Hotopf M. How equitable are psychological therapy services in South East London now? A comparison of referrals to a new psychological therapy service with participants in a psychiatric morbidity survey in the same London borough. Social Psychiatry and Psychiatric Epidemiology: The International Journal for Research in Social and Genetic Epidemiology and Mental Health Services. 2014;49(12):1893-902.

67. Butler M, Delvi A, Mujic F, Broad S, Pauli L, Pollak TA, et al. Reduced Activity in an Inpatient Liaison Psychiatry Service During the First Wave of the COVID-19 Pandemic: Comparison With 2019 Data and Characterization of the SARS-CoV-2 Positive Cohort. Frontiers in Psychiatry. 2021;12:619550.

68. Byrne A, Barber R, Lim CH. Impact of the COVID-19 pandemic – a mental health service perspective. Progress in Neurology and Psychiatry. 2021;25(2):27-33b.

69. Carr MJ, Ashcroft DM, Kontopantelis E, While D, Awenat Y, Cooper J, et al. Clinical management following self-harm in a UK-wide primary care cohort. Journal of Affective Disorders. 2016;197:182-8.

70. Carruthers E, Oakeshott P. Refugee and asylum seeker usage of primary care: medical student survey at two inner-city general practices. Education for Primary Care. 2019;30(4):248-50.

71. Chaplin R, Farquharson L, Clapp M, Crawford M. Comparison of access, outcomes and experiences of older adults and working age adults in psychological therapy. International Journal of Geriatric Psychiatry. 2015;30(2):178-84.

72. Chen S, She R, Qin P, Kershenbaum A, Fernandez-Egea E, Nelder JR, et al. The Medium-Term Impact of COVID-19 Lockdown on Referrals to Secondary Care Mental Health Services: A Controlled Interrupted Time Series Study. Frontiers in Psychiatry. 2020;11(1307).

73. Clement S, Williams P, Farrelly S, Hatch SL, Schauman O, Jeffery D, et al. Mental Health-Related Discrimination as a Predictor of Low Engagement With Mental Health Services. Psychiatric Services. 2015;66(2):171-6.

74. Colling C, Evans L, Broadbent M, Chandran D, Craig TJ, Kolliakou A, et al. Identification of the delivery of cognitive behavioural therapy for psychosis (CBTp) using a cross-sectional sample from electronic health records and open-text information in a large UK-based mental health case register. BMJ Open. 2017;7(7):e015297.

75. Cullen AE, Bowers L, Khondoker M, Pettit S, Achilla E, Koeser L, et al. Factors associated with use of psychiatric intensive care and seclusion in adult inpatient mental health services. Epidemiology and Psychiatric

Sciences. 2018;27(1):51-61.

76. Dagnan D, Rodhouse C, Thwaites R, Hatton C. Improving Access to Psychological Therapies (IAPT) services outcomes for people with learning disabilities: national data 2012-2013 to 2019-2020. Cognitive Behaviour Therapist. 2022;15:15.

77. Das-Munshi J, Bhugra D, Crawford MJ. Ethnic minority inequalities in access to treatments for schizophrenia and schizoaffective disorders: findings from a nationally representative cross-sectional study. BMC Medicine. 2018;16(1):55.

78. Day E, Shah R, Taylor RW, Marwood L, Nortey K, Harvey J, et al. A retrospective examination of care pathways in individuals with treatment-resistant depression. BJPsych Open. 2021;7(3):e101.

79. Delgadillo J, Asaria M, Ali S, Gilbody S. On poverty, politics and psychology: the socioeconomic gradient of mental healthcare utilisation and outcomest. British Journal of Psychiatry. 2016;209(5):431-2.

80. Delgadillo J, Farnfield A, North A. Social inequalities in the demand, supply and utilisation of psychological treatment. Counselling & Psychotherapy Research. 2018;18(2):114-21.

81. Di Bona L, Saxon D, Barkham M, Dent-Brown K, Parry G. Predictors of patient non-attendance at Improving Access to Psychological Therapy services demonstration sites. Journal of Affective Disorders. 2014;169:157-64.

82. Domoney J, Howard LM, Abas M, Broadbent M, Oram S. Mental health service responses to human trafficking: a qualitative study of professionals' experiences of providing care. BMC Psychiatry. 2015;15(1):289.

83. Dorrington S, Carr E, Stevelink S, Ashworth M, Broadbent M, Madan I, et al. Access to mental healthcare in the year after first fit note: a longitudinal study of linked clinical records. BMJ Open. 2021;11(11):e044725.

84. Ellis SJ, Bailey L, McNeil J. Trans People's Experiences of Mental Health and Gender Identity Services: A UK Study. Journal of Gay & Lesbian Mental Health. 2015;19(1):4-20.

85. Fernández de la Cruz L, Llorens M, Jassi A, Krebs G, Vidal-Ribas P, Radua J, et al. Ethnic inequalities in the use of secondary and tertiary mental health services among patients with obsessive–compulsive disorder. British Journal of Psychiatry. 2015;207(6):530-5.

86. Firth N, Delgadillo J, Kellett S, Lucock M. The influence of socio-demographic similarity and difference on adequate attendance of group psychoeducational cognitive behavioural therapy. Psychotherapy Research. 2020;30(3):362-74.

87. Forrester A, Samele C, Slade K, Craig T, Valmaggia L. Demographic and clinical characteristics of 1092 consecutive police custody mental health referrals. Journal of Forensic Psychiatry & Psychology. 2017;28(3):295-312.

88. Gajwani R, Parsons H, Birchwood M, Singh SP. Ethnicity and detention: are Black and minority ethnic (BME) groups disproportionately detained under the Mental Health Act 2007? Social Psychiatry and Psychiatric Epidemiology. 2016;51(5):703-11.

89. Gazard B, Chui Z, Harber-Aschan L, MacCrimmon S, Bakolis I, Rimes K, et al. Barrier or stressor? The role of discrimination experiences in health service use. BMC Public Health. 2018;18(1):1354.

90. Giebel CM, Clarkson P, Challis D. Demographic and clinical characteristics of UK military veterans attending a psychological therapies service. Psychiatric Bulletin. 2014;38(6):270-5.

91. Giebel C, Corcoran R, Goodall M, Campbell N, Gabbay M, Daras K, et al. Do people living in disadvantaged circumstances receive different mental health treatments than those from less disadvantaged backgrounds? BMC Public Health. 2020;20(1):10.

92. Gnan GH, Rahman Q, Ussher G, Baker D, West E, Rimes KA. General and LGBTQ-specific factors associated with mental health and suicide risk among LGBTQ students. Journal of Youth Studies. 2019;22(10):1393-408.

93. Godier-McBard LR, Cable G, Wood AD, Fossey M. Gender differences in barriers to mental healthcare for UK military veterans: a preliminary investigation. BMJ Military Health. 2022;168(1):70-5.

94. Harwood H, Rhead R, Chui Z, Bakolis I, Connor L, Gazard B, et al. Variations by ethnicity in referral and treatment pathways for IAPT service users in South London. Psychological Medicine. 2021:1-12.

95. Holman D. What help can you get talking to somebody?' Explaining class differences in the use of talking treatments. Sociology of Health & Illness. 2014;36(4):531-48.

96. Hopkin G, Chaplin L, Slade K, Craster L, Valmaggia L, Samele C, et al. Differences between homeless and non-homeless people in a matched sample referred for mental health reasons in police custody. International Journal of Social Psychiatry. 2020;66(6):576-83.

97. Jakobowitz S, Bebbington P, McKenzie N, Iveson R, Duffield G, Kerr M, et al. Assessing needs for psychiatric treatment in prisoners: 2. Met and unmet need. Social Psychiatry and Psychiatric Epidemiology. 2017;52(2):231-40.

98. Jankovic J, Parsons J, Jovanović N, Berrisford G, Copello A, Fazil Q, et al. Differences in access and utilisation of mental health services in the perinatal period for women from ethnic minorities—a population-based study. BMC Medicine. 2020;18(1):245.

99. Kapadia D, Nazroo J, Tranmer M. Ethnic differences in women's use of mental health services: do social networks play a role? Findings from a national survey. Ethnicity & Health. 2018;23(3):293-306.

100. Kirkbride JB, Hameed Y, Wright L, Russell K, Knight C, Perez J, et al. Waiting time variation in Early Intervention Psychosis services: longitudinal evidence from the SEPEA naturalistic cohort study. Social Psychiatry and Psychiatric Epidemiology. 2017;52(5):563-74.

101. Kothari R, White D, Craster L, Vicianova E, Dennard S, Bailey F, et al. The impact of integrating mental health services within a prison setting. Mental Health Review Journal. 2022;27(2):146-57.

102. Lappin JM, Heslin M, Jones PB, Doody GA, Reininghaus UA, Demjaha A, et al. Outcomes following first-episode psychosis - Why we should intervene early in all ages, not only in youth. The Australian and New Zealand Journal of Psychiatry. 2016;50(11):1055-63.

103. Lawrence V, McCombie C, Nikolakopoulos G, Morgan C. Navigating the mental health system: Narratives of identity and recovery among people with psychosis across ethnic groups. Social Science & Medicine. 2021;279:9.

104. Lawrence V, McCombie C, Nikolakopoulos G, Morgan C. Ethnicity and power in the mental health system: experiences of white British and black Caribbean people with psychosis. Epidemiology and Psychiatric

Sciences. 2021;30:e12.

105. Leavey G, McGrellis S, Forbes T, Thampi A, Davidson G, Rosato M, et al. Improving mental health pathways and care for adolescents in transition to adult services (IMPACT): a retrospective case note review of social and clinical determinants of transition. Social Psychiatry and Psychiatric Epidemiology. 2019;54(8):955-63.

106. Livanou MI, Lane R, D'Souza S, Singh SP. A retrospective case note review of young people in transition from adolescent medium secure units to adult services. Journal of Forensic Practice. 2020;22(3):161-72.

107. Livanou M, Singh SP, Liapi F, Furtado V. Mapping transitional care pathways among young people discharged from adolescent forensic medium secure units in England. Medicine, Science and the Law. 2020;60(1):45-53.

108. Livanou M, D'Souza S, Lane R, La Plante B, Singh SP. Challenges and Facilitators During Transitions from Adolescent Medium Secure Units to Adult Services in England: Interviews with Mental Healthcare Professionals. Administration and Policy in Mental Health and Mental Health Services Research. 2021;48(6):1089-104.

109. Maconick L, Sheridan Rains L, Jones R, Lloyd-Evans B, Johnson S. Investigating geographical variation in the use of mental health services by area of England: a cross-sectional ecological study. BMC Health Services Research. 2021;21(1):951.

110. Majid M, Tadros M, Tadros G, Singh S, Broome MR, Upthegrove R. Young people who self-harm: a prospective 1-year follow-up study. Social Psychiatry and Psychiatric Epidemiology. 2016;51(2):171-81.

111. Manescu EA, Robinson EJ, Henderson C. Attitudinal and demographic factors associated with seeking help and receiving antidepressant medication for symptoms of common mental disorder. BMC Psychiatry. 2020;20(1):579.

112. Mankiewicz PD, Reid J, Hughes EA, Attard A. Management of demographic equality of access to family intervention for psychosis in specialist community mental healthcare teams. British Journal of Healthcare Management. 2021;27(8):1-11.

113. Mann F, Fisher HL, Major B, Lawrence J, Tapfumaneyi A, Joyce J, et al. Ethnic variations in compulsory detention and hospital admission for psychosis across four UK Early Intervention Services. BMC Psychiatry. 2014;14(1):256.

114. Mansour R, Tsamakis K, Rizos E, Perera G, Das-Munshi J, Stewart R, et al. Late-life depression in people from ethnic minority backgrounds: Differences in presentation and management. Journal of Affective Disorders. 2020;264:340-7.

115. Mark KM, Leightley D, Pernet D, Murphy D, Stevelink SAM, Fear NT. Identifying Veterans Using Electronic Health Records in the United Kingdom: A Feasibility Study. Healthcare. 2020;8(1):13.

116. Matthew Prina A, Marioni RE, Hammond GC, Jones PB, Brayne C, Dening T. Improving access to psychological therapies and older people: findings from the Eastern Region. Behaviour Research and Therapy. 2014;56(100):75-81.

117. McKenzie N, Killaspy H, Jakobowitz S, Faranak H, Bebbington P. Assessing needs for psychiatric treatment in prisoners: 3. Comparison of care received by black and minority ethnic prisoners and by white prisoners. Social Psychiatry and Psychiatric Epidemiology. 2019;54(7):883-6.

118. McNamara N, Coyne I, Ford T, Paul M, Singh S, McNicholas F. Exploring social identity change during mental healthcare transition. European Journal of Social Psychology. 2017;47(7):889-903.

119. Meddings S, Walsh L, Patmore L, McKenzie KLE, Holmes S. To what extent does Sussex Recovery College reflect its community? An equalities and diversity audit. Mental Health and Social Inclusion. 2019;23(3):136-44.

120. Mercer L, Evans LJ, Turton R, Beck A. Psychological Therapy in Secondary Mental Health Care: Access and Outcomes by Ethnic Group. Journal of Racial and Ethnic Health Disparities. 2019;6(2):419-26.

121. Mirza A, Birtel MD, Pyle M, Morrison AP. Cultural Differences in Psychosis: The Role of Causal Beliefs and Stigma in White British and South Asians. Journal of Cross-Cultural Psychology. 2019;50(3):441-59.

122. Moore L, Jayaweera H, Redshaw M, Quigley M. Migration, ethnicity and mental health: evidence from mothers participating in the Millennium Cohort Study. Public Health. 2019;171:66-75.

123. Morgan J. Does a cluster always equal a cluster? Geographical variation of cluster populations. Psychiatric Bulletin. 2014;38(6):294-8.

124. Morgan C, Fearon P, Lappin J, Heslin M, Donoghue K, Lomas B, et al. Ethnicity and long-term course and outcome of psychotic disorders in a UK sample: the AESOP-10 study. British Journal of Psychiatry. 2017;211(2):88-94.

125. Nicholson L, Hotchin H. The relationship between area deprivation and contact with community intellectual disability psychiatry. Journal of Intellectual Disability Research. 2015;59(5):487-92.

126. Nilforooshan R, Benson L, Gage H, Williams P, Zoha M, Warner J. Comparison of service utilisation and costs of working age adults and older adults receiving treatment for psychosis and severe non-psychotic conditions in England: implications for commissioning. International Journal of Geriatric Psychiatry. 2017;32(1):110-5.

127. Oates LL, Firth N. Deprivation, access and outcomes in health psychology treatment. Mental Health Review Journal. 2020;25(2):139-51.

128. Oduola S, Craig TKJ, Das-Munshi J, Bourque F, Gayer-Anderson C, Morgan C. Compulsory admission at first presentation to services for psychosis: does ethnicity still matter? Findings from two population-based studies of first episode psychosis. Social Psychiatry and Psychiatric Epidemiology. 2019;54(7):871-81.

129. Pettit S, Qureshi A, Lee W, Stirzaker A, Gibson A, Henley W, et al. Variation in referral and access to new psychological therapy services by age: an empirical quantitative study. British Journal of General Practice. 2017;67(660):e453-e9.

130. Prady SL, Pickett KE, Gilbody S, Petherick ES, Mason D, Sheldon TA, et al. Variation and ethnic inequalities in treatment of common mental disorders before, during and after pregnancy: combined analysis of routine and research data in the Born in Bradford cohort. BMC Psychiatry. 2016;16(1):99.

131. Reichert A, Jacobs R. Socioeconomic inequalities in duration of untreated psychosis: evidence from administrative data in England. Psychological Medicine. 2018;48(5):822-33.

132. Rhead RD, Woodhead C, Ahmad G, Das-Munshi J, McManus S, Hatch SL. A comparison of single and intersectional social identities associated with discrimination and mental health service use: data from the 2014 Adult Psychiatric Morbidity Survey in England. Social Psychiatry and Psychiatric Epidemiology. 2022;57(10):2049-63.

133. Ride J, Kasteridis P, Gutacker N, Aragon Aragon MJ, Jacobs R. Healthcare Costs for People with Serious Mental Illness in England: An Analysis of Costs Across Primary Care, Hospital Care, and Specialist Mental Healthcare. Applied Health Economics and Health Policy. 2020;18(2):177-88.

134. Saini P, Chopra J, Hanlon CA, Boland JE. A Case Series Study of Help-Seeking among Younger and Older Men in Suicidal Crisis. International Journal of Environmental Research and Public Health. 2021;18(14):14.

135. Singh SP, Brown L, Winsper C, Gajwani R, Islam Z, Jasani R, et al. Ethnicity and pathways to care during first episode psychosis: the role of cultural illness attributions. BMC Psychiatry. 2015;15:287.

136. Sizmur S, McCulloch A. Differences in treatment approach between ethnic groups. Mental Health Review Journal. 2016;21(2):73-84.

137. Smyth N, Buckman JEJ, Naqvi SA, Aguirre E, Cardoso A, Pilling S, et al. Understanding differences in mental health service use by men: an intersectional analysis of routine data. Social Psychiatry and Psychiatric Epidemiology. 2022;57(10):2065-77.

138. Steeg S, Carr M, Trefan L, Ashcroft D, Kapur N, Nielsen E, et al. Primary care clinical management following self-harm during the first wave of COVID-19 in the UK: population-based cohort study. BMJ Open. 2022;12(2):e052613.

139. Thomas F, Hansford L, Ford J, Wyatt K, McCabe R, Byng R. How accessible and acceptable are current GP referral mechanisms for IAPT for low-income patients? Lay and primary care perspectives. Journal of Mental Health. 2020;29(6):706-11.

140. Tseliou F, Johnson S, Major B, Rahaman N, Joyce J, Lawrence J, et al. Gender differences in one-year outcomes of first-presentation psychosis patients in inner-city UK Early Intervention Services. Early Intervention in Psychiatry. 2017;11(3):215-23.

141. Tucker S, Wilberforce M, Brand C, Abendstern M, Crook A, Jasper R, et al. Community mental health teams for older people: variations in case mix and service receipt (I). International Journal of Geriatric Psychiatry. 2015;30(6):595-604.

142. Tyler N, Miles HL, Karadag B, Rogers G. An updated picture of the mental health needs of male and female prisoners in the UK: prevalence, comorbidity, and gender differences. Social Psychiatry and Psychiatric Epidemiology. 2019;54(9):1143-52.

143. Valmaggia LR, Byrne M, Day F, Broome MR, Johns L, Howes O, et al. Duration of untreated psychosis and need for admission in patients who engage with mental health services in the prodromal phase. British Journal of Psychiatry. 2015;207(2):130-4.

144. Volkert J, Andreas S, Harter M, Dehoust MC, Sehner S, Suling A, et al. Predisposing, enabling, and need factors of service utilization in the elderly with mental health problems. International Psychogeriatrics. 2018;30(7):1027-37.

145. Walters K, Falcaro M, Freemantle N, King M, Ben-Shlomo Y. Sociodemographic inequalities in the management of depression in adults aged 55 and over: an analysis of English primary care data. Psychological Medicine. 2018;48(9):1504-13.

146. Watson A, Mellotte H, Hardy A, Peters E, Keen N, Kane F. The digital divide: factors impacting on uptake of remote therapy in a South London psychological therapy service for people with psychosis. Journal of Mental Health. 2022;31(6):825-32.

147. Weich S, McBride O, Twigg L, Duncan C, Keown P, Crepaz-Keay D, et al. Variation in compulsory psychiatric inpatient admission in England: a cross-classified, multilevel analysis. The Lancet Psychiatry. 2017;4(8):619-26.

148. White J, Gutacker N, Jacobs R, Mason A. Hospital admissions for severe mental illness in England: Changes in equity of utilisation at the small area level between 2006 and 2010. Social Science & Medicine. 2014;120:243-51.

149. Wilberforce M, Tucker S, Brand C, Abendstern M, Jasper R, Stewart K, et al. Community mental health teams for older people: variations in case mix and service receipt (II). International Journal of Geriatric Psychiatry. 2015;30(6):605-13.

150. Yasmin-Qureshi S, Ledwith S. Beyond the barriers: South Asian women's experience of accessing and receiving psychological therapy in primary care. Journal of Public Mental Health. 2021;20(1):3-14.

151. Liberati E, Richards N, Parker J, Willars J, Scott D, Boydell N, et al. Remote care for mental health: qualitative study with service users, carers and staff during the COVID-19 pandemic. BMJ Open. 2021;11(4):e049210.

152. Wiginton JM, Maksut JL, Murray SM, Augustinavicius JL, Kall M, Delpech V, et al. Brief report: HIV-related healthcare stigma/discrimination and unmet needs among persons living with HIV in England and Wales. Preventive Medicine Reports. 2021;24:101580.
