# Supplementary material for: Understanding inequalities in access to adult mental health services in the UK: a systematic mapping review
Source: BMC Health Serv Res. 2023 Sep 29;23:1042. doi: 10.1186/s12913-023-10030-8 (PMC10542667; doi:10.1186/s12913-023-10030-8)
Supplement: Supplementary file 4 — Additional file 4: Table S4. Summary of key findings associated with dimensions of inequality [file 12913_2023_10030_MOESM4_ESM.docx]

**Additional file 4**

**Table S4.** Summary of key findings associated with dimensions of inequality

| **Dimension of inequality** | **Studies reported data for dimension** | **Studies did not report data for dimension** | **Studies which only included specific population groups** | **Differences in levels of access to mental health services (e.g. referrals, service use)** | **Differences in ways of accessing mental health services (e.g. pathways, referral sources)** | **Barriers to accessing mental health services – using Levesque framework** |
| --- | --- | --- | --- | --- | --- | --- |
| Age | 117 (77%) | 35 (23%) | - Young adults (n=11) - Older adults (n=4) | - **no differences in access** – by age (1-6) - **MH costs** – increased for younger adults (7, 8), increased for older adults (7-9) - **formal MH help-seeking** – less likely for younger adults (10), more likely for middle age adults (11), less likely for older adults (12), more likely for older adults (13) - **referrals to MH services** – those referred during COVID-19 were younger (14), more likely to be of working age (15), vary by age (16-19), males more likely to be younger at referral (20) - **access to MH services** – lower for younger adults (21), higher for younger adults (22-27), lower for older adults (28), IAPT patients more likely to be younger (29) - **engagement with MH treatment** – lower for younger adults (30), higher for older adults (28, 31) | - **referral source** – GP-referred and self-referred IAPT patients more likely to be younger (29) , older adults less likely to be referred by GP and more likely to self-refer (31) - **compulsory MH treatment –** those aged over 35 (32) and aged 40-54 (33) more likely to be subjected to an MHA section, risk of compulsory admission higher in those aged 18-35 (34) - **waiting times** – older adults lower waiting times for MH treatment (28, 31, 35) - **service provision –** significant variation for older adults (36, 37) - **service delivery** – remote MH care accessed by younger adults (38) | - **ability to perceive** – difficulty in recognising a MH problem (39, 40), eligibility (40, 41), illness identity (42), trust (43) - **ability to seek** – awareness of services (42), autonomy (39, 42), self-reliance (40), stigma and discrimination (39-42, 44) - **ability to reach** – availability of services (43, 45, 46), flexibility (43, 46), technology (38, 47, 48) transition (43, 45, 46, 49-51) - **ability to engage** – appropriateness of services (36, 37, 41) continuity of care (41, 43, 46), joint working (43, 46), family/carer involvement (41, 42, 50) |
| Disability | 27 (18%) | 125 (82%) | - Learning / intellectual disabilities (n=3) - Long-term conditions (n=1) - Physical health conditions (n=1) - Deaf people (n=1) - Living with HIV (n=1) | - **no differences in access** – by disability (4, 26, 52, 53) - **MH costs** – increased for people with physical health conditions (7, 8) - **formal MH help-seeking** – more likely for people with long-term conditions (11) - **referrals to MH services** – increased for people with existing conditions following COVID-19 (15) - **access to MH services** – lower for people in receipt of fit note (26), lower disclosure of disability (27) - **engagement with MH treatment** – lower IAPT uptake and higher IAPT dropout rate for people with learning disabilities (52) - **unmet MH needs** – high for people with disabilities (54), high for people living with HIV (55) | - | - **ability to percieve** – eligibility (55, 56) - **ability to seek** – awareness of services (56, 57), stigma and discrimination (55, 56, 58) - **ability to reach** – availability of services (58, 59), flexibility (58), language and communication (57), technology (47, 59, 60), transport (47, 58) - **ability to engage** – appropriateness of services (56, 57), coordination of MH and physical health care (58), family/carer involvement (56, 57) |
| Education | 36 (24%) | 116 (76%) | - University students (n=6) | - **no differences in access** – by education (13, 39) - **formal MH help-seeking** – less likely for people with no qualifications (11), less likely for people with higher educational levels (13, 61), more likely for people with higher educational levels (12) - **referrals to MH services** – males more likely to have less qualifications at referral (20) - **access to MH services** – lower for people with no qualifications (62), higher for university students (61, 63), ethnic minority MH patients more likely to be educated at GCSE or above (64) | - | - **ability to seek** – stigma and discrimination (44, 65) |
| Gender and sex | 125 (82%) | 27 (18%) | - Females (n=11) - Males (n=7) - Transgender (n=1) | - **no differences in access** – by gender or sex (1, 3, 4, 6, 13-15, 20, 23, 24, 26, 30, 32, 33, 35, 38, 66-68) - **MH costs** – increased for males (8) - **formal MH help-seeking** – less likely for males (11, 61), more likely for females (10) - **referrals to MH services** – lower for males (49), higher for females (16) - **access to MH services –** lower for males (3, 61) (39), access to secondary MH services higher for males (26), IAPT patients more likely to be male (29), higher for females (27, 44, 62, 63, 69, 70), access to IAPT services higher for females (26), lower for transgender people (27), high for transgender people (63, 71) - **unmet MH needs** – high for males (70, 72) | - **referral source** – GP-referred IAPT patients more likely to be male, and no differences by gender in self-referred IAPT patients (29), males more likely to be referred by social/criminal justice services (68) - **referral destination** – males more likely to be referred to inpatient and emergency services than outpatient (68) - **compulsory treatment** – more likely for males (34, 73), more likely for females (20) | - **ability to percieve** – difficulty in recognising MH problem (74), eligibility (71), trust (69, 74) - **ability to seek** – awareness of services (74), stigma and discrimination (61, 65, 69) - **ability to reach** – technology (38) - **ability to engage** – appropriateness of services (71, 74) |
| Occupation | 57 (38%) | 95 (62%) | - UK Armed Forces / veterans (n=11) - Doctors / GPs (n=3) | - **no differences in access** – by employment (13, 35, 75) - **formal MH help-seeking** – more likely for unemployed people (11) - **referrals to MH services –** males less likely to be employed full-time at referral (20) - **access to MH services** – lower for employed people (76), higher for unemployed people (44, 49, 62, 77, 78), IAPT patients more likely to be unemployed or full-time homemakers/ carers and less likely to be students or retired (29), Black MH patients more likely to be unemployed (79) - **engagement with MH treatment** – less likely for unemployed people (30), higher risk of disengaging for unemployed males (75) | - **referral source** – GP-referred IAPT patients more likely to be unemployed or full-time homemakers/carers and less likely to be students or retired (29), self-referred IAPT patients more likely to be unemployed and less likely to be students or retired (29), UK armed forces personnel benefit from availability of a self-referral pathway (80) - **referral destination** – unemployed males higher risk of being deemed unsuitable by MH services, and higher risk of being referred elsewhere (75), veterans use mainstream NHS services rather than veteran specific services (69) - **compulsory treatment –** more likely for unemployed people (81) - **waiting times** – MH treatment waiting times longer for unemployed people (6, 25, 82) and students (6) | - **ability to perceive** – difficulty in recognising MH problem (83-89), eligibility (84, 89), trust (69, 84, 88-90) - **ability to seek** – awareness of services (83-85, 88, 89), autonomy (86), stigma and discrimination (44, 69, 83-91) - **ability to reach** – availability of services (83, 85, 87-89), flexibility (83, 85, 91), language and communication (87), social support (86, 89), time (83, 84, 88, 91), - **ability to engage** – appropriateness of services (83, 88, 89) |
| Place of residence | 51 (33%) | 101 (67%) | - Homeless (n=1) | - **no differences in access** – by area of living (12, 18, 33, 35), or by accommodation type (6, 35) - **MH costs** - increased for those living alone or in communal households (7), decreased for greater distance between GP and MH services (8) - **formal MH help-seeking** – more likely for those living alone (12) - **referrals to MH services** – lower for homeless people (53) - **access to MH services –** lower for homeless people (53), higher for those living alone (36), higher for those with housing problems (21), male MH patients more likely to be living with relatives (20), female MH patients more likely to be living alone (20), geographical variations in access (1, 39, 78) - **engagement with MH treatment** – lower for homeless people (53) | - **compulsory treatment** – more likely for those living alone (81), more likely for those living in supported accommodation (32) - **service provision** – significant geographical variations in MH resources (92) | - **ability to percieve** – unable to prioritise MH (93) - **ability to seek** – awareness of services (93) - **ability to reach** – location (7), privacy (94), safety (47), technology (47, 93), transport (93) - **ability to engage** – appropriateness of services (93) |
| Pregnancy and maternity | 8 (5%) | 144 (95%) | - Women in the pre-natal / post-natal period (n=6 - Mothers (n=1) | - | - | - **ability to percieve** – difficulty in recognising MH problem (95) - **ability to seek** – awareness of services (96), stigma and discrimination (95, 96) - **ability to reach** – flexibility (95) - **ability to engage** – appropriateness of services (94, 95) |
| Race, ethnicity, culture, and language | 116 (76%) | 38 (24%) | - Ethnic minority groups (n=17) | - **no differences in access** – by ethnicity (1-3, 6, 11, 14, 30, 33, 38, 53, 76, 92, 97-100), or migration status (11, 68, 77) - **MH costs** – increased for White people (8), increased for ethnic minority groups (7) - **formal MH help-seeking** – more likely for Black university students (101) - **access to MH services** – less likely for ethnic minority groups (13, 24, 26, 27, 44, 62, 64, 77, 102-107), less likely for migrants (62, 102, 108), more likely for White people (23, 109), increased access for White people following COVID-19 (15), IAPT patients more likely to be White (29) - **engagement with MH treatment** – higher risk of disengagement for ethnic minority males (75) - **unmet MH needs** – high for ethnic minority groups (24, 64) | - **referral source** – GP-referred IAPT patients more likely to be White (29), no differences by ethnicity in self-referred IAPT patients (29), Black people higher rates of criminal justice system involvement (32, 68, 79, 110-112), lower MH access via GP for migrants (62) - **referral destination** – ethnic minority males higher risk of being deemed unsuitable by MH services, and higher risk of being referred elsewhere (75), ethnic minority groups more likely to be referred to inpatient and emergency services than outpatient (68) - **compulsory treatment** – more likely for Black people (34, 79, 81, 105, 110-113), more likely for ethnic minority groups (32, 33) - **waiting times** – lower for ethnic minority groups (35), ethnic minority groups more likely to present later to MH services (82) | - **ability to percieve** – difficulty in recognising MH problem (114-120), eligibility (121), illness attributions (109, 111, 118, 120, 122), illness identity (123), trust (115-117, 122, 124-128) - **ability to seek** – autonomy (123, 125), awareness of services (116, 117, 119, 127-129), culture (60, 96, 114-117, 119, 121-125, 127, 128, 130, 131), stigma and discrimination (44, 65, 96, 97, 109, 114-117, 119-124, 126-131) - **ability to reach** – availability of services (115, 119, 121, 128, 132, 133), flexibility (121), language and communication (47, 96, 114, 116, 119, 131, 132), social support (114, 117, 119, 124, 127, 130), technology (38, 133) - **ability to engage** – appropriateness of services (60, 96, 117, 119, 121, 125, 127, 129, 130, 133), continuity of care (116, 126), family/carer involvement (124, 126, 131), power (114, 119, 124, 125) |
| Religion | 12 (8%) | 140 (92%) | - | - **no differences in access** – by religion (3) - **access to MH services** – higher for Christian people (27), lower for non-religious people (27) - **engagement with MH treatment** – higher risk of disengagement for Muslim males (75), lower risk of disengagement for Christian males (75) | - | - **ability to perceive** – illness attributions (111, 117) - **ability to seek** – culture (131) |
| Sexual orientation | 15 (10%) | 137 (90%) | - Sexual minority groups (n=3) | - **access to MH services** –lower for sexual minority groups (27), higher for sexual minority groups (44, 63), lower for heterosexual people (101) - **engagement with MH treatment** – higher risk of disengagement for sexual minority males (75) | - | - **ability to percieve** – disclosure of sexual orientation (67, 134) - **ability to seek** – stigma and discrimination (44, 63, 67, 134) - **ability to reach** – social support (63) - **ability to engage** – appropriateness of services (67, 94, 134) |
| Social capital | 6 (4%) | 146 (96%) | - | - **no differences in access** – by social capital (11, 12) - **formal MH help-seeking** – more likely for increased sense of belonging (101), more likely for increased social support (10) - **access to MH services** – lower for those with adequate social support (77) | - | - **ability to reach** – social support (10, 101) |
| Socio-economic status | 39 (26%) | 113 (74%) | - Low income patients (n=1) | - **no differences in access** – by socio-economic status (16, 18, 26, 35, 75, 76, 128, 135) - **MH costs** – increased for people from more deprived areas (7, 8) - **formal MH help-seeking** – more likely for lower income patients (11) - **referrals to MH services** – less likely for people from more deprived areas (136, 137) - **access to MH services** – lower for people from more deprived areas (30, 92), higher for people from more deprived areas (44, 78, 138-140) - **engagement with MH treatment** – higher risk of disengagement for males from more deprived areas (75) | - **referral source** – GP-referred IAPT patients less likely to be in reciept of benefits (29), no differences for self-referred IAPT patients on benefit status (29) - **compulsory treatment –** more likely for people from more deprived areas (34) - **waiting times** – lower for people from least deprived areas (6) | - **ability to perceive** – eligibility (141) - **ability to seek** – self-reliance (141) - **ability to reach** – technology (47, 48, 60, 142), availability of services (141) - **ability to engage** – appropriateness of services (141, 143) |
| *Contact with criminal justice system | 8 (5%) | 144 (95%) | - Prisoners (n=5) - Probationers (n=1) | - **access to MH services –** high for prisoners (64, 70, 73) - **unmet MH needs** – high for prisoners (64, 72) | - | - **ability to perceive** – trust (97), unable to prioritise MH (144) - **ability to seek** – self-reliance (97), stigma and discrimination (97) - **ability to reach** – availability of services (72, 97, 144) - **ability to engage** – appropriateness of services (72, 144) |
| *Refugees and asylum seekers | 3 (2%) | 149 (98%) | - Refugees and asylum seekers (n=3) | - | - **referral destination** – despite refugee/asylum seekers presenting at GP, very few referred to IAPT or MH services (145) | - **ability to perceive** – health beliefs and practices (146) - **ability to seek** – stigma and discrimination (146) - **ability to reach** – availability of services (132), language and communication (132, 146) |
| *Trafficked people and street sex workers | 3 (2%) | 149 (98%) | - Trafficked people (n=2) - Street sex workers (n=1) | - | - | - **ability to percieve** – disclosure of being trafficked (147), eligibility (148), trust (148) - **ability to reach** – availability of services (147-149) - **ability to engage** – appropriateness of services (147-149) |
| *Marital or relationship status | 35 (23%) | 117 (77%) | - | - **no differences in access** – by marital or relationship status (6, 13, 23, 35) - **formal MH help-seeking** – more likely for non-married/non-cohabiting people (11) - **referrals to MH services** - males more likely to be single at referral (20) - **access to MH services** – increased for non-cohabiting people following COVID-19 (15), Black MH patients more likely to be single (79) - **engagement with MH treatment** – IAPT uptake less likely for those previously cohabiting (1) | - **compulsory treatment** – more likely for single people (33) | - |

* COVID-19, coronavirus 19; GP, general practitioner; HIV, human immunodeficiency virus; IAPT, improving access to psychological therapies; MH, mental health; MHA, Mental Health Act; NHS, national health service; UK, United Kingdom

**Included studies references**

1. Di Bona L, Saxon D, Barkham M, Dent-Brown K, Parry G. Predictors of patient non-attendance at Improving Access to Psychological Therapy services demonstration sites. Journal of Affective Disorders. 2014;169:157-64.

2. Kothari R, White D, Craster L, Vicianova E, Dennard S, Bailey F, et al. The impact of integrating mental health services within a prison setting. Mental Health Review Journal. 2022;27(2):146-57.

3. Mankiewicz PD, Reid J, Hughes EA, Attard A. Management of demographic equality of access to family intervention for psychosis in specialist community mental healthcare teams. British Journal of Healthcare Management. 2021;27(8):1-11.

4. Morgan J. Does a cluster always equal a cluster? Geographical variation of cluster populations. The Psychiatric Bulletin. 2014;38(6):294-8.

5. Saini P, Chopra J, Hanlon CA, Bol, JE. A Case Series Study of Help-Seeking among Younger and Older Men in Suicidal Crisis. International Journal of Environmental Research and Public Health. 2021;18(14):14.

6. Reichert A, Jacobs R. Socioeconomic inequalities in duration of untreated psychosis: evidence from administrative data in England. Psychological Medicine. 2018;48(5):822-33.

7. Anselmi L, Everton A, Shaw R, Suzuki W, Burrows J, Weir R, et al. Estimating local need for mental healthcare to inform fair resource allocation in the NHS in England: cross-sectional analysis of national administrative data linked at person level. British Journal of Psychiatry. 2020;216(6):338-44.

8. Ride J, Kasteridis P, Gutacker N, Aragon MJA, Jacobs R. Healthcare Costs for People with Serious Mental Illness in England: An Analysis of Costs Across Primary Care, Hospital Care, and Specialist Mental Healthcare. Applied Health Economics and Health Policy. 2020;18(2):177-88.

9. Nilforooshan R, Benson L, Gage H, Williams P, Zoha M, Warner J. Comparison of service utilisation and costs of working age adults and older adults receiving treatment for psychosis and severe non‐psychotic conditions in england: Implications for commissioning. International Journal of Geriatric Psychiatry. 2017;32(1):110-5.

10. Stevelink SAM, Jones N, Jones M, Dyball D, Khera CK, Pernet D, et al. Do serving and ex-serving personnel of the UK armed forces seek help for perceived stress, emotional or mental health problems? European Journal of Psychotraumatology. 2019;10(1):1556552.

11. Brown JSL, Evans-Lacko S, Aschan L, Henderson MJ, Hatch SL, Hotopf M. Seeking informal and formal help for mental health problems in the community: a secondary analysis from a psychiatric morbidity survey in South London. BMC Psychiatry. 2014;14(1):275.

12. Bu FF, Mak HW, Fancourt D. Rates and predictors of uptake of mental health support during the COVID-19 pandemic: an analysis of 26,720 adults in the UK in lockdown. Social Psychiatry and Psychiatric Epidemiology. 2021;56(12):2287-97.

13. Gondek D, Kirkbride JB. Predictors of mental health help-seeking among polish people living the United Kingdom. Bmc Health Services Research. 2018;18:12.

14. Butler M, Delvi A, Mujic F, Broad S, Pauli L, Pollak TA, et al. Reduced Activity in an Inpatient Liaison Psychiatry Service During the First Wave of the COVID-19 Pandemic: Comparison With 2019 Data and Characterization of the SARS-CoV-2 Positive Cohort. Frontiers in Psychiatry. 2021;12.

15. Chen SQ, She R, Qin P, Kershenbaum A, Fern, ez-Egea E, et al. The Medium-Term Impact of COVID-19 Lockdown on Referrals to Secondary Care Mental Health Services: A Controlled Interrupted Time Series Study. Frontiers in Psychiatry. 2020;11:11.

16. Oates LL, Firth N. Deprivation, access and outcomes in health psychology treatment. Mental Health Review Journal. 2020;25(2):139-51.

17. Steeg S, Carr M, Trefan L, Ashcroft D, Kapur N, Nielsen E, et al. Primary care clinical management following self-harm during the first wave of COVID-19 in the UK: population-based cohort study. Bmj Open. 2022;12(2):8.

18. Walters K, Falcaro M, Freemantle N, King M, Ben-Shlomo Y. Sociodemographic inequalities in the management of depression in adults aged 55 and over: an analysis of English primary care data. Psychological Medicine. 2018;48(9):1504-13.

19. Pettit S, Qureshi A, Lee W, Byng R, Gibson A, Stirzaker A, et al. Variation in referral and access to new psychological therapy services by age: An empirical quantitative study. British Journal of General Practice. 2017;67(660):e453-e9.

20. Tseliou F, Johnson S, Major B, Rahaman N, Joyce J, Lawrence J, et al. Gender differences in one-year outcomes of first-presentation psychosis patients in inner-city UK Early Intervention Services. Early Intervention in Psychiatry. 2017;11(3):215-23.

21. Majid M, Tadros M, Tadros G, Singh S, Broome MR, Upthegrove R. Young people who self-harm: a prospective 1-year follow-up study. Social Psychiatry and Psychiatric Epidemiology. 2016;51(2):171-81.

22. Lappin JM, Heslin M, Jones PB, Doody GA, Reininghaus UA, Demjaha A, et al. Outcomes following first-episode psychosis - Why we should intervene early in all ages, not only in youth. The Australian and New Zealand Journal of Psychiatry. 2016;50(11):1055-63.

23. Colling C, Evans L, Broadbent M, Ch, ran D, Craig TJ, et al. Identification of the delivery of cognitive behavioural therapy for psychosis (CBTp) using a cross-sectional sample from electronic health records and open-text information in a large UK-based mental health case register. BMJ Open. 2017;7(7):e1002141.

24. Sizmur S, McCulloch A. Differences in treatment approach between ethnic groups. Mental Health Review Journal. 2016;21(2):73-84.

25. Day E, Shah R, Taylor RW, Marwood L, Nortey K, Harvey J, et al. A retrospective examination of care pathways in individuals with treatment-resistant depression. Bjpsych Open. 2021;7(3):11.

26. Dorrington S, Carr E, Stevelink S, Ashworth M, Broadbent M, Madan I, et al. Access to mental healthcare in the year after first fit note: a longitudinal study of linked clinical records. BMJ Open. 2021;11(11):e044725.

27. Meddings S, Walsh L, Patmore L, McKenzie KLE, Holmes S. To what extent does Sussex Recovery College reflect its community? An equalities and diversity audit. Mental Health & Social Inclusion. 2019;23(3):136-44.

28. Chaplin R, Farquharson L, Clapp M, Crawford M. Comparison of access, outcomes and experiences of older adults and working age adults in psychological therapy. International journal of geriatric psychiatry. 2015;30(2):178-84.

29. Brown J, Ferner H, Wingrove J, Aschan L, Hatch S, Hotopf M. How equitable are psychological therapy services in South East London now? A comparison of referrals to a new psychological therapy service with participants in a psychiatric morbidity survey in the same London borough. Social Psychiatry & Psychiatric Epidemiology. 2014;49(12):1893-902.

30. Firth N, Delgadillo J, Kellett S, Lucock M. The influence of socio-demographic similarity and difference on adequate attendance of group psychoeducational cognitive behavioural therapy. Psychotherapy Research. 2020;30(3):362-74.

31. Matthew Prina A, Marioni RE, Hammond GC, Jones PB, Brayne C, Dening T. Improving access to psychological therapies and older people: Findings from the Eastern Region. Behaviour Research & Therapy. 2014;56:75-81.

32. Gajwani R, Parsons H, Birchwood M, Singh SP. Ethnicity and detention: are Black and minority ethnic (BME) groups disproportionately detained under the Mental Health Act 2007? Social Psychiatry and Psychiatric Epidemiology. 2016;51(5):703-11.

33. Watson J, Daley S. The use of section 135(1) of the Mental Health Act in a London borough. Mental Health Review Journal. 2015;20(3):133-43.

34. Weich S, McBride O, Twigg L, Duncan C, Keown P, Crepaz-Keay D, et al. Variation in compulsory psychiatric inpatient admission in England: a cross-classified, multilevel analysis. Lancet Psychiatry. 2017;4(8):619-26.

35. Kirkbride J, Hameed Y, Wright L, Russell K, Knight C, Perez J, et al. Waiting time variation in Early Intervention Psychosis services: longitudinal evidence from the SEPEA naturalistic cohort study. Social Psychiatry & Psychiatric Epidemiology. 2017;52(5):563-74.

36. Wilberforce M, Tucker S, Br, C, Abendstern M, Jasper R, et al. Community mental health teams for older people: Variations in case mix and service receipt (II). International Journal of Geriatric Psychiatry. 2015;30(6):605-13.

37. Tucker S, Wilberforce M, Br, C, Abendstern M, Crook A, et al. Community mental health teams for older people: Variations in case mix and service receipt (I). International Journal of Geriatric Psychiatry. 2015;30(6):595-604.

38. Watson A, Mellotte H, Hardy A, Peters E, Keen N, Kane F. The digital divide: factors impacting on uptake of remote therapy in a South London psychological therapy service for people with psychosis. Journal of Mental Health. 2021.

39. Volkert J, Andreas S, Harter M, Dehoust MC, Sehner S, Suling A, et al. Predisposing, enabling, and need factors of service utilization in the elderly with mental health problems. International Psychogeriatrics. 2018;30(7):1027-37.

40. Salaheddin K, Mason B. Identifying barriers to mental health help-seeking among young adults in the UK: a cross-sectional survey. British Journal of General Practice. 2016;66(651):E686-E92.

41. Berry K, Sheardown J, Pabbineedi U, Haddock G, Cross C, Brown LJE. Barriers and facilitators to accessing psychological therapies for severe mental health difficulties in later life. Behavioural and Cognitive Psychotherapy. 2020;48(2):216-28.

42. McNamara N, Coyne I, Ford T, Paul M, Singh S, McNicholas F. Exploring social identity change during mental healthcare transition. European Journal of Social Psychology. 2017;47(7):889-903.

43. Butterworth S, Singh SP, Birchwood M, Islam Z, Munro ER, Vostanis P, et al. Transitioning care-leavers with mental health needs: "they set you up to fail!'. Child and Adolescent Mental Health. 2017;22(3):138-47.

44. Rhead RD, Woodhead C, Ahmad G, Das-Munshi J, McManus S, Hatch SL. A comparison of single and intersectional social identities associated with discrimination and mental health service use: data from the 2014 Adult Psychiatric Morbidity Survey in England. Social Psychiatry and Psychiatric Epidemiology. 2022:15.

45. Livanou M, Singh SP, Liapi F, Furtado V. Mapping transitional care pathways among young people discharged from adolescent forensic medium secure units in England. Medicine, Science and the Law. 2019;60(1):45-53.

46. van der Kamp J. The transition between mental health services in Scotland. Mental Health Review Journal. 2018;23(1):12-24.

47. Liberati E, Richards N, Parker J, Willars J, Scott D, Boydell N, et al. Remote care for mental health: Qualitative study with service users, carers and staff during the COVID-19 pandemic. BMJ Open. 2021;11(4):49210.

48. Shah P, Hardy J, Birken M, Foye U, Rowan Olive R, Nyikavar, et al. What has changed in the experiences of people with mental health problems during the COVID-19 pandemic: a coproduced, qualitative interview study. Social Psychiatry & Psychiatric Epidemiology. 2022;57(6):1291-303.

49. Leavey G, McGrellis S, Forbes T, Thampi A, Davidson G, Rosato M, et al. Improving mental health pathways and care for adolescents in transition to adult services (IMPACT): a retrospective case note review of social and clinical determinants of transition. Social Psychiatry and Psychiatric Epidemiology. 2019;54(8):955-63.

50. Livanou M, D'Souza S, Lane R, La Plante B, Singh SP. Challenges and Facilitators During Transitions from Adolescent Medium Secure Units to Adult Services in England: Interviews with Mental Healthcare Professionals. Administration and policy in mental health. 2021;48(6):1089-104.

51. Livanou MI, Lane R, D'Souza S, Singh SP. A retrospective case note review of young people in transition from adolescent medium secure units to adult services. Journal of Forensic Practice. 2020;22(3):161-72.

52. Dagnan D, Rodhouse C, Thwaites R, Hatton C. Improving Access to Psychological Therapies (IAPT) services outcomes for people with learning disabilities: national data 2012-2013 to 2019-2020. Cognitive Behaviour Therapist. 2022;15:15.

53. Hopkin G, Chaplin L, Slade K, Craster L, Valmaggia L, Samele C, et al. Differences between homeless and non-homeless people in a matched sample referred for mental health reasons in police custody. International Journal of Social Psychiatry. 2020;66(6):576-83.

54. Sakellariou D, Rotarou ES. Access to healthcare for men and women with disabilities in the UK: secondary analysis of cross-sectional data. Bmj Open. 2017;7(8):9.

55. Wiginton JM, Maksut JL, Murray SM, Augustinavicius JL, Kall M, Delpech V, et al. Brief report: HIV-related healthcare stigma/discrimination and unmet needs among persons living with HIV in England and Wales. Preventive Medicine Reports. 2021;24:101580.

56. Chinn D, Abraham E. Using 'candidacy' as a framework for understanding access to mainstream psychological treatment for people with intellectual disabilities and common mental health problems within the English Improving Access to Psychological Therapies service. Journal of Intellectual Disability Research. 2016;60(6):571-82.

57. Reader D, Foulkes H, Robinson C. Investigating barriers to mental health care experienced by the Deaf community in North Wales. Mental Health Nursing. 2017;37(3):14-9.

58. Carroll S, Moss‐Morris R, Hulme K, Hudson J. Therapists' perceptions of barriers and facilitators to uptake and engagement with therapy in long‐term conditions. British Journal of Health Psychology. 2021;26(2):307-24.

59. Gregson N, le-Phillips C, Delaney C. Delivering Psychological Services for People with Learning Disabilities during the Covid-19 Pandemic: The Experiences of Psychologists in the UK. Journal of Mental Health Research in Intellectual Disabilities. 2022;15(2):168-96.

60. Gillard S, Dare C, Hardy J, Nyikavar, a P, Rowan Olive R, et al. Experiences of living with mental health problems during the COVID-19 pandemic in the UK: a coproduced, participatory qualitative interview study. Social Psychiatry & Psychiatric Epidemiology. 2021;56(8):1447-57.

61. Ennis E, McLafferty M, Murray E, Lapsley C, Bjourson T, Armour C, et al. Readiness to change and barriers to treatment seeking in college students with a mental disorder. Journal of Affective Disorders. 2019;252:428-34.

62. Bhavsar V, Jannesari S, McGuire P, MacCabe JH, Das-Munshi J, Bhugra D, et al. The association of migration and ethnicity with use of the Improving Access to Psychological Treatment (IAPT) programme: a general population cohort study. Social Psychiatry and Psychiatric Epidemiology. 2021;56(11):1943-56.

63. Gnan GH, Rahman Q, Ussher G, Baker D, West E, Rimes KA. General and LGBTQ-specific factors associated with mental health and suicide risk among LGBTQ students. Journal of Youth Studies. 2019;22(10):1393-408.

64. McKenzie N, Killaspy H, Jakobowitz S, Faranak H, Bebbington P. Assessing needs for psychiatric treatment in prisoners: 3. Comparison of care received by black and minority ethnic prisoners and by white prisoners. Social Psychiatry and Psychiatric Epidemiology. 2019;54(7):883-6.

65. Dockery L, Jeffery D, Schauman O, Williams P, Farrelly S, Bonnington O, et al. Stigma- and non-stigma-related treatment barriers to mental healthcare reported by service users and caregivers. Psychiatry Research. 2015;228(3):612-9.

66. Forrester A, Samele C, Slade K, Craig T, Valmaggia L. Demographic and clinical characteristics of 1092 consecutive police custody mental health referrals. Journal of Forensic Psychiatry & Psychology. 2017;28(3):295-312.

67. Foy AAJ, Morris D, Fern, es V, Rimes KA. LGBQ+ adults' experiences of Improving Access to Psychological Therapies and primary care counselling services: informing clinical practice and service delivery. Cognitive Behaviour Therapist. 2019;12:23.

68. Chui Z, Gazard B, MacCrimmon S, Harwood H, Downs J, Bakolis I, et al. Inequalities in referral pathways for young people accessing secondary mental health services in south east London. European Child & Adolescent Psychiatry. 2021;30(7):1113-28.

69. Godier-McBard LR, Cable G, Wood AD, Fossey M. Gender differences in barriers to mental healthcare for UK military veterans: a preliminary investigation. BMJ Military Health. 2022;168(1):70-5.

70. Tyler N, Miles HL, Karadag B, Rogers G. An updated picture of the mental health needs of male and female prisoners in the UK: prevalence, comorbidity, and gender differences. Social Psychiatry and Psychiatric Epidemiology. 2019;54(9):1143-52.

71. Ellis SJ, Bailey L, McNeil J. Trans People's Experiences of Mental Health and Gender Identity Services: A UK Study. Journal of Gay & Lesbian Mental Health. 2015;19(1):4-20.

72. Jakobowitz S, Bebbington P, McKenzie N, Iveson R, Duffield G, Kerr M, et al. Assessing needs for psychiatric treatment in prisoners: 2. Met and unmet need. Social Psychiatry and Psychiatric Epidemiology. 2017;52(2):231-40.

73. Bebbington P, Jakobowitz S, McKenzie N, Killaspy H, Iveson R, Duffield G, et al. Assessing needs for psychiatric treatment in prisoners: 1. Prevalence of disorder. Social psychiatry and psychiatric epidemiology. 2017;52(2):221-9.

74. Sagar-Ouriaghli I, Brown JSL, Tailor V, Godfrey E. Engaging male students with mental health support: a qualitative focus group study. Bmc Public Health. 2020;20(1):14.

75. Smyth N, Buckman JEJ, Naqvi SA, Aguirre E, Cardoso A, Pilling S, et al. Understanding differences in mental health service use by men: an intersectional analysis of routine data. Social Psychiatry and Psychiatric Epidemiology. 2022:13.

76. Manescu EA, Robinson EJ, Henderson C. Attitudinal and demographic factors associated with seeking help and receiving antidepressant medication for symptoms of common mental disorder. Bmc Psychiatry. 2020;20(1):11.

77. Kapadia D, Nazroo J, Tranmer M. Ethnic differences in women's use of mental health services: do social networks play a role? Findings from a national survey. Ethnicity & Health. 2018;23(3):293-306.

78. Maconick L, Rains LS, Jones R, Lloyd-Evans B, Johnson S. Investigating geographical variation in the use of mental health services by area of England: a cross-sectional ecological study. Bmc Health Services Research. 2021;21(1):10.

79. Morgan C, Fearon P, Lappin J, Heslin M, Donoghue K, Lomas B, et al. Ethnicity and long-term course and outcome of psychotic disorders in a UK sample: the AESOP-10 study. British Journal of Psychiatry. 2017;211(2):88-94.

80. Kennedy I, Whybrow D, Jones N, Sharpley J, Greenberg N. A service evaluation of self-referral to military mental health teams. Occupational Medicine-Oxford. 2016;66(5):394-8.

81. Oduola S, Craig TKJ, Das-Munshi J, Bourque F, Gayer-Anderson C, Morgan C. Compulsory admission at first presentation to services for psychosis: does ethnicity still matter? Findings from two population-based studies of first episode psychosis. Social Psychiatry and Psychiatric Epidemiology. 2019;54(7):871-81.

82. Valmaggia LR, Byrne M, Day F, Broome MR, Johns L, Howes O, et al. Duration of untreated psychosis and need for admission in patients who engage with mental health services in the prodromal phase. British Journal of Psychiatry. 2015;207(2):130-4.

83. Brooks SK, Gerada C, Chalder T. The specific needs of doctors with mental health problems: qualitative analysis of doctor-patients' experiences with the Practitioner Health Programme. Journal of Mental Health. 2017;26(2):161-6.

84. Daniels J, Ingram J, Pease A, Wainwright E, Beckett K, Iyadurai L, et al. The COVID-19 Clinician Cohort (CoCCo) Study: Empirically Grounded Recommendations for Forward-Facing Psychological Care of Frontline Doctors. International Journal of Environmental Research and Public Health. 2021;18(18):18.

85. Mellotte H, Murphy D, Rafferty L, Greenberg N. Pathways into mental health care for UK veterans: a qualitative study. European Journal of Psychotraumatology. 2017;8(1):11.

86. Murphy D, Hunt E, Luzon O, Greenberg N. Exploring positive pathways to care for members of the UK Armed Forces receiving treatment for PTSD: a qualitative study. European Journal of Psychotraumatology. 2014;5:8.

87. Rafferty LA, Wessely S, Stevelink SAM, Greenberg N. The journey to professional mental health support: a qualitative exploration of the barriers and facilitators impacting military veterans' engagement with mental health treatment. European Journal of Psychotraumatology. 2019;10(1):14.

88. Spiers J, Kessler D, Leggett N, Taylor AK, Thornton G, Buszewicz M, et al. Barriers, facilitators, and survival strategies for GPs seeking treatment for distress: A qualitative study. British Journal of General Practice. 2017;67(663):e700-e8.

89. Williamson V, Pearson EJ, Shevlin M, Karatzias T, Macmanus D, Murphy D. Experiences of Veterans with ICD-11 Complex PTSD in Engaging with Services. Journal of Loss & Trauma. 2021;26(2):166-78.

90. Fertout M, Jones N, Keeling M, Greenberg N. Mental health stigmatisation in deployed UK Armed Forces: a principal components analysis. Journal of the Royal Army Medical Corps. 2015;161:69-76.

91. Williamson V, Greenberg N, Stevelink SAM. Perceived stigma and barriers to care in UK Armed Forces personnel and veterans with and without probable mental disorders. BMC psychology. 2019;7(1):75.

92. Delgadillo J, Farnfield A, North A. Social inequalities in the demand, supply and utilisation of psychological treatment. Counselling & Psychotherapy Research. 2018;18(2):114-21.

93. Adams EA, Parker J, Jablonski T, Kennedy J, Tasker F, Hunter D, et al. A Qualitative Study Exploring Access to Mental Health and Substance Use Support among Individuals Experiencing Homelessness during COVID-19. International Journal of Environmental Research and Public Health. 2022;19(6):3459.

94. Harrop E, Goss S, Farnell D, Longo M, Byrne A, Barawi K, et al. Support needs and barriers to accessing support: Baseline results of a mixed-methods national survey of people bereaved during the COVID-19 pandemic. Palliative Medicine. 2021;35(10):1985-97.

95. Millett L, Taylor BL, Howard LM, Bick D, Stanley N, Johnson S. Experiences of Improving Access to Psychological Therapy Services for Perinatal Mental Health Difficulties: a Qualitative Study of Women's and Therapists' Views. Behavioural and Cognitive Psychotherapy. 2018;46(4):421-36.

96. Watson H, Soltani H. Perinatal mental ill health: the experiences of women from ethnic minority groups. British Journal of Midwifery. 2019;27(10):642-8.

97. McGrath K, Shaw J, Farquharson L. Barriers to accessing psychological treatment for medium to high risk male young offenders. Journal of Forensic Psychiatry & Psychology. 2020;31(4):596-612.

98. Byrne M, Codjoe L, Morgan C, Stahl D, Day F, Fearon P, et al. The relationship between ethnicity and service access, treatment uptake and the incidence of psychosis among people at ultra high risk for psychosis. Psychiatry Research. 2019;272:618-27.

99. Cullen AE, Bowers L, Khondoker M, Pettit S, Achilla E, Koeser L, et al. Factors associated with use of psychiatric intensive care and seclusion in adult inpatient mental health services. Epidemiology and Psychiatric Sciences. 2018;27(1):51-61.

100. Mansour R, Tsamakis K, Rizos E, Perera G, Das-Munshi J, Stewart R, et al. Late-life depression in people from ethnic minority backgrounds: Differences in presentation and management. Journal of Affective Disorders. 2020;264:340-7.

101. Bryant A, Cook A, Egan H, Wood J, Mantzios M. Help-seeking behaviours for mental health in higher education. Journal of Further and Higher Education. 2022;46(4):522-34.

102. Moore L, Jayaweera H, Redshaw M, Quigley M. Migration, ethnicity and mental health: evidence from mothers participating in the Millennium Cohort Study. Public Health. 2019;171:66-75.

103. Prady SL, Pickett KE, Gilbody S, Petherick ES, Mason D, Sheldon TA, et al. Variation and ethnic inequalities in treatment of common mental disorders before, during and after pregnancy: combined analysis of routine and research data in the Born in Bradford cohort. BMC Psychiatry. 2016;16(1):99.

104. Das-Munshi J, Bhugra D, Crawford MJ. Ethnic minority inequalities in access to treatments for schizophrenia and schizoaffective disorders: findings from a nationally representative cross-sectional study. BMC Medicine. 2018;16(1):55.

105. Jankovic J, Parsons J, Jovanovic N, Berrisford G, Copello A, Fazil Q, et al. Differences in access and utilisation of mental health services in the perinatal period for women from ethnic minorities-a population-based study. Bmc Medicine. 2020;18(1):12.

106. Fernández de la Cruz L, Llorens M, Jassi A, Krebs G, Vidal-Ribas P, Radua J, et al. Ethnic inequalities in the use of secondary and tertiary mental health services among patients with obsessive–compulsive disorder. The British Journal of Psychiatry. 2015;207(6):530-5.

107. Mercer L, Evans LJ, Turton R, Beck A. Psychological Therapy in Secondary Mental Health Care: Access and Outcomes by Ethnic Group. Journal of Racial and Ethnic Health Disparities. 2019;6(2):419-26.

108. Harwood H, Rhead R, Chui Z, Bakolis I, Connor L, Gazard B, et al. Variations by ethnicity in referral and treatment pathways for IAPT service users in South London. Psychological Medicine. 2021:1-12.

109. Mirza A, Birtel MD, Pyle M, Morrison AP. Cultural Differences in Psychosis: The Role of Causal Beliefs and Stigma in White British and South Asians. Journal of Cross-Cultural Psychology. 2019;50(3):441-59.

110. Mann F, Fisher HL, Major B, Lawrence J, Tapfumaneyi A, Joyce J, et al. Ethnic variations in compulsory detention and hospital admission for psychosis across four UK Early Intervention Services. BMC Psychiatry. 2014;14(1):256.

111. Singh SP, Brown L, Winsper C, Gajwani R, Islam Z, Jasani R, et al. Ethnicity and pathways to care during first episode psychosis: the role of cultural illness attributions. Bmc Psychiatry. 2015;15:8.

112. Ajnakina O, Lally J, Di Forti M, Kolliakou A, Gardner-Sood P, Lopez-Morinigo J, et al. Patterns of illness and care over the 5 years following onset of psychosis in different ethnic groups; the GAP-5 study. Social Psychiatry and Psychiatric Epidemiology. 2017;52(9):1101-11.

113. Bansal N, Bhopal R, Netto G, Lyons D, Steiner MFC, Sashidharan SP. Disparate patterns of hospitalisation reflect unmet needs and persistent ethnic inequalities in mental health care: the Scottish health and ethnicity linkage study. Ethnicity & Health. 2014;19(2):217-39.

114. Arday J. Understanding mental health: What are the issues for black and ethnic minority students at University? Social Sciences. 2018;7(10).

115. Bailey NV, Tribe R. A qualitative study to explore the help-seeking views relating to depression among older Black Caribbean adults living in the UK. International Review of Psychiatry. 2021;33(1):113-8.

116. Islam Z, Rabiee F, Singh SP. Black and Minority Ethnic Groups' Perception and Experience of Early Intervention in Psychosis Services in the United Kingdom. Journal of Cross-Cultural Psychology. 2015;46(5):737-53.

117. Sancho TN, Larkin M. "We need to slowly break down this barrier": understanding the barriers and facilitators that Afro-Caribbean undergraduates perceive towards accessing mental health services in the UK. Journal of Public Mental Health. 2020;19(1):63-81.

118. Fernandez de la Cruz L, Kolvenbach S, Vidal-Ribas P, Jassi A, Llorens M, Patel N, et al. Illness perception, help-seeking attitudes, and knowledge related to obsessive-compulsive disorder across different ethnic groups: a community survey. Social Psychiatry and Psychiatric Epidemiology. 2016;51(3):455-64.

119. Memon A, Taylor K, Mohebati LM, Sundin J, Cooper M, Scanlon T, et al. Perceived barriers to accessing mental health services among black and minority ethnic (BME) communities: a qualitative study in Southeast England. BMJ Open. 2016;6(11):9.

120. Yeung EYW, Irvine F, Ng SM, Tsang KMS. How people from Chinese backgrounds make sense of and respond to the experiences of mental distress: Thematic analysis. Journal of Psychiatric and Mental Health Nursing. 2017;24(8):589-99.

121. Yasmin-Qureshi S, Ledwith S. Beyond the barriers: South Asian women's experience of accessing and receiving psychological therapy in primary care. Journal of Public Mental Health. 2020;20(1):3-14.

122. Moller N, Burgess V, Jogiyat Z. Barriers to counselling experienced by British South Asian women: A thematic analysis exploration. Counselling & Psychotherapy Research. 2016;16(3):201-10.

123. Lawrence V, McCombie C, Nikolakopoulos G, Morgan C. Navigating the mental health system: Narratives of identity and recovery among people with psychosis across ethnic groups. Social Science & Medicine. 2021;279:9.

124. Pilav S, De Backer K, Easter A, Silverio SA, Sundaresh S, Roberts S, et al. A qualitative study of minority ethnic women's experiences of access to and engagement with perinatal mental health care. BMC pregnancy and childbirth. 2022;22(1):421.

125. Lawrence V, McCombie C, Nikolakopoulos G, Morgan C. Ethnicity and power in the mental health system: experiences of white British and black Caribbean people with psychosis. Epidemiology and Psychiatric Sciences. 2021;30:7.

126. Rabiee F, Smith P. Understanding mental health and experience of accessing services among African and African Caribbean Service users and carers in Birmingham, UK. Diversity and Equality in Health and Care. 2014;11(2):125-34.

127. Thompson RM, Stone BV, Tyson PJ. Mental health support needs within Gypsy, Roma, and Traveller communities: a qualitative study. Mental Health and Social Inclusion. 2022;26(2):144-55.

128. Ogueji IA, Okoloba MM. Seeking Professional Help for Mental Illness: A Mixed-Methods Study of Black Family Members in the UK and Nigeria. Psychological Studies. 2022:1-14.

129. Bellesi G, Jeraj S, Manley J, Tekes S, Basit H, McNulty N. Why do black caribbean women benefit less from talking therapies? A pilot study in an inner London IAPT service. Clinical Psychology Forum. 2020;2020(326):24-9.

130. Kanakam N. Therapists' Experiences of Working with Ethnic Minority Females with Eating Disorders: A Qualitative Study. Culture Medicine and Psychiatry. 2022;46(2):414-34.

131. Simkhada B, Vahdaninia M, vanTeijlingen E, Blunt H. Cultural issues on accessing mental health services in Nepali and Iranian migrants communities in the UK. International Journal of Mental Health Nursing. 2021;30(6):1610-9.

132. Khanom A, Alanazy W, Couzens L, Evans BA, Fagan L, Fogarty R, et al. Asylum seekers' and refugees' experiences of accessing health care: a qualitative study. BJGP Open. 2021;5(6).

133. Pilav S, Easter A, Silverio SA, De Backer K, Sundaresh S, Roberts S, et al. Experiences of Perinatal Mental Health Care among Minority Ethnic Women during the COVID-19 Pandemic in London: A Qualitative Study. International Journal of Environmental Research and Public Health. 2022;19(4):15.

134. Morris DDA, Fern, es V, Rimes KA. Sexual minority service user perspectives on mental health treatment barriers to care and service improvements. International Review of Psychiatry. 2022.

135. Nicholson L, Hotchin H. The relationship between area deprivation and contact with community intellectual disability psychiatry. Journal of Intellectual Disability Research. 2015;59(5):487-92.

136. Carr MJ, Ashcroft DM, Kontopantelis E, While D, Awenat Y, Cooper J, et al. Clinical management following self-harm in a UK-wide primary care cohort. Journal of Affective Disorders. 2016;197:182-8.

137. Delgadillo J, Asaria M, Ali S, Gilbody S. On poverty, politics and psychology: the socioeconomic gradient of mental healthcare utilisation and outcomest. British Journal of Psychiatry. 2016;209(5):431-2.

138. Gazard B, Chui Z, Harber-Aschan L, MacCrimmon S, Bakolis I, Rimes K, et al. Barrier or stressor? The role of discrimination experiences in health service use. BMC Public Health. 2018;18(1):1354.

139. White J, Gutacker N, Jacobs R, Mason A. Hospital admissions for severe mental illness in England: Changes in equity of utilisation at the small area level between 2006 and 2010. Social Science & Medicine. 2014;120:243-51.

140. Giebel C, Corcoran R, Goodall M, Campbell N, Gabbay M, Daras K, et al. Do people living in disadvantaged circumstances receive different mental health treatments than those from less disadvantaged backgrounds? BMC Public Health. 2020;20(1):10.

141. Thomas F, Hansford L, Ford J, Wyatt K, McCabe R, Byng R. How accessible and acceptable are current GP referral mechanisms for IAPT for low-income patients? Lay and primary care perspectives. Journal of Mental Health. 2020;29(6):706-11.

142. Liberati E, Richards N, Parker J, Willars J, Scott D, Boydell N, et al. Qualitative study of candidacy and access to secondary mental health services during the COVID-19 pandemic. Social Science and Medicine. 2022;296:114711.

143. Holman D. What help can you get talking to somebody?' Explaining class differences in the use of talking treatments. Sociology of Health & Illness. 2014;36(4):531-48.

144. Plugge E, Pari AAA, Maxwell J, Holl, S. When prison is “easier”: probationers’ perceptions of health and wellbeing. International Journal of Prisoner Health. 2014;10(1):38-46.

145. Carruthers E, Oakeshott P. Refugee and asylum seeker usage of primary care: medical student survey at two inner-city general practices. Education for Primary Care. 2019;30(4):248-50.

146. Paudyal P, Tattan M, Cooper MJF. Qualitative study on mental health and well-being of Syrian refugees and their coping mechanisms towards integration in the UK. BMJ Open. 2021;11(8):9.

147. Domoney J, Howard LM, Abas M, Broadbent M, Oram S. Mental health service responses to human trafficking: a qualitative study of professionals’ experiences of providing care. BMC Psychiatry. 2015;15(1):289.

148. Potter LC, Horwood J, Feder G. Access to healthcare for street sex workers in the UK: perspectives and best practice guidance from a national cross-sectional survey of frontline workers. Bmc Health Services Research. 2022;22(1):11.

149. Williamson V, Borschmann R, Zimmerman C, Howard LM, Stanley N, Oram S. Responding to the health needs of trafficked people: A qualitative study of professionals in England and Scotland. Health & Social Care in the Community. 2019;28(1):173-81.
